# The fus test: a promising tool for evaluating fundamental motor skills in children and adolescents

**DOI:** 10.1186/s12889-023-16843-w

**Published:** 2023-10-03

**Authors:** Hubert Makaruk, Jared M. Porter, E. Kipling Webster, Beata Makaruk, Anna Bodasińska, Janusz Zieliński, Paweł Tomaszewski, Marta Nogal, Paulina Szyszka, Marcin Starzak, Marcin Śliwa, Michał Banaś, Michał Biegajło, Agata Chaliburda, Dariusz Gierczuk, Bogusz Suchecki, Bartosz Molik, Jerzy Sadowski

**Affiliations:** 1 Faculty of Physical Education and Health in Biala Podlaska, Józef Piłsudski University of Physical Education in Warsaw, Warsaw, Poland; 2 Department of Kinesiology, Recreation, and Sport Studies, University of Tennessee, Knoxville, USA; 3 Faculty of Physical Education, Józef Piłsudski University of Physical Education in Warsaw, Warsaw, Poland; 4 Faculty of Rehabilitation, Józef Piłsudski University of Physical Education in Warsaw, Warsaw, Poland

**Keywords:** Motor competence, Fundamental movement skills, Physical education, Physical activity

## Abstract

Fundamental motor skills (FMS) are essential for enjoyable, confident and skillful participation in physical activity across the lifespan. Due to the alarming low level of FMS proficiency in children and adolescents worldwide, the development of motor competency is an urgent issue for physical education. The promotion and implementation of a systematic process of teaching and learning FMS should be a physical education priority. Accordingly, effective assessment tools for evaluating FMS should be adopted or developed. Because FMS assessment for both children and adolescents need further effective solutions, the primary aim of this study was to develop the new age-related test of FMS (Fundamental Motor Skills in Sport test, *in Polish*: Test Fundamentalnych Umiejętności Ruchowych w Sporcie, FUS). The secondary aim of this study was to establish validity and inter-rater, intra-rater, test-retest reliabilities and internal consistency of the FUS test. The FUS test involves six sport skill-based tasks: hurdling, jumping rope, forward roll, ball bouncing, throwing and catching a ball, and kicking and stopping a ball. Two hundred sixty-four Polish students in grades 1–3 (7–9 yrs; *n* = 81), 4–6 (10–12 yrs; *n* = 89) and 7–8 (13–14 yrs; *n* = 94), including 139 girls and 125 boys completed the FUS test. The content validity index for all items was notably high. Both inter-rater and intra-rater reliability showed substantial to almost perfect agreement, with observed agreements for FUS skills between 78.5 and 93.1%. Ball bouncing had a moderate correlation with the forward roll and throwing and catching, while other correlations were low or insignificant. ICC values, ranging from 0.95 to 0.97, confirmed excellent test-retest reliability. The results of our study provide evidence that the FUS test is valid, reliable, and feasible to administer in school settings. Therefore, this tool test has the potential to support deliberate practice and improve motor competence by providing a standardized and structured approach to measuring FMS among school-aged children and adolescents.

**Supplementary Information:**

The online version contains supplementary material available at 10.1186/s12889-023-16843-w.

## Key points

The FUS test is a valid, reliable, feasible and user-friendly tool for FMS assessment of children and adolescent in school settings.

The FMS utilized in the FUS test is based on a wide range of FMS involved in various sports and lifetime activities that are popular across the world. This comprehensive approach to motor skill development allows the FUS test to assess a broad range of skills and provide a holistic evaluation of a child’s motor function. By incorporating a variety of activities and movements, the FUS test can also help children develop a diverse set of motor skills that can be applied to different physical activities throughout their lives.

The FUS test may enhance motivation and support deliberate practice of FMS by providing a structured framework for assessment and feedback. The test allows children to track their progress over time and identify areas for improvement, which can help to build self-efficacy and encourage continued engagement in physical activity.

**Supplementary Information:**

The online version contains supplementary material available at 10.1186/s12889-023-16843-w.

## Introduction

### Understanding fundamental motor skills (FMS)

One of the main goals of physical education (PE) is to develop health, well-being and skillful people who are able to enjoy the benefits of physical activity and sport throughout their lives. With this in mind, PE curriculums should emphasize motor competence, because like physical fitness and enjoyment of physical activity, developing motor competence facilitates the achievement of PE goals [1, 2]. Motor competence refers to an individual’s degree of proficiency in performing basic, but essential movement patterns [3]. While motor competence is considered more of a global term that is comprised of multiple facets of skilled behavior, early movement behaviors that establish the foundation for later movement experiences are termed fundamental motor skills or fundamental movement skills (FMS) [4, 5]. FMS are foundational skills, such as jumping, kicking, and striking, that serve as precursors for more specialized, complex skills used in organized and non-organized games and sports [3]. FMS are often categorized as locomotor skills (e.g., running, jumping), object control or manipulative skills (e.g., throwing, kicking) and balance or stability skills (e.g., one-foot balance, body rolling) [4, 6]. A common misconception is that children learn FMS naturally, spontaneously and unassisted; however, proper learning of FMS is often a long and arduous process of acquisition that requires support from PE teachers, sport coaches, and parents to teach, practice, and reinforce these skills [7]. The foundation for FMS is established early in life, with children often possessing the potential to master most of these skills by the age of 6–8 [6].

### Potential benefits of enhanced FMS

The acquisition of FMS is a critical determinant of a healthy childhood. Children with a high level of FMS can confidently and with enjoyment participate in a wide range of physical activities [8, 9]. The list of benefits associated with high levels of FMS in children is very long, with improvements in cardiorespiratory fitness [10, 11], higher engagement in physical activity [12, 13], and lower levels of adiposity [8, 14]. It also presumes that children with high FMS proficiency possess higher levels of cardiorespiratory fitness and perceived sports competence [11]. However, it is important to note that the relationship between FMS and physical activity is not yet sufficiently recognized. Although Stodden et al. [15] suggested that motor competence may be a catalyst for physical activity in middle to late childhood, Robinson et al. [8] found a lack of studies specifically addressing the relationship between motor competence and physical activity among adolescents. In turn, Barnett et al. [14] reassessed the connections theorized by Stodden et al. [15] via experimental or longitudinal studies, finding the evidence linking motor competence to physical activity to be largely inconclusive. The potential as well as the intricacies of this still unrecognized issue underscore the need for further research for a deeper understanding of FMS.

### Current practices in FMS assessment

Typically, FMS are assessed by product and/or process-oriented tests. A product-oriented approach focuses on quantitative analysis of an individual’s motor performance (e.g., how far a child can jump or throw a ball). A process-oriented assessment is qualitative and is concerned with how movements were performed during a task. For example, a PE teacher using the process-oriented approach may evaluate how the legs or arms were positioned in relation to the torso during a jump [4]. Due to the greater complexity of conducting a qualitative assessment, it is believed that process-oriented assessment often requires more knowledge and training in its administration [16], and assessment becomes more sensitive to assessor experience and subjectivity [17]. Therefore, some authors question the feasibility of using process-oriented testing in a school setting, pointing to limitations such as the need for the evaluator to obtain expertise in the correct movement pattern of a skill [16]. Additionally, most teachers have limited time for assessment during a lesson unit, causing another obstacle for using a more time-consuming process-oriented approach. Nevertheless, process-oriented assessment seems to provide a more complete view of FMS proficiency, as it allows to separately evaluate individual components of a movement pattern, which as a whole, demonstrates the degree of mastery of a given skill. In contrast, a product-oriented assessment only reports the resultant outcome of a performance. This is probably one of the reasons why process-oriented assessment is more commonly used in FMS testing [4]. Some researchers suggest that using both methods simultaneously may provide a more holistic measurement of FMS [4, 18].

One of the most widely used tools, the Test of Gross Motor Development (TGMD), is a process-oriented test that contains product-oriented components. This standardized and norm-referenced tool has been validated to assess and evaluate the gross motor skills of children between the ages of 3 and 10 years [19, 20]. The latest version of the TGMD-third edition (TGMD-3) [19], measures 13 gross motor skills, including the run, gallop, hop, skip, jumping forward, slide, two-hand strike, one-hand strike, stationary dribble, catch, kick, overhand throw, and underhand throw. Each skill is evaluated on 3–5 performance criteria that reflect the appropriate movement pattern. When the performance complies with the criterion, the participant is awarded a score of “1” for each trial, when the criterion is not met, a score of “0” is awarded. Scores are the total number of points obtained in two trials assessing each skill, and skills are usually assessed collectively in two subcategories: locomotor or object control (termed Ball Skills in the TGMD-3). The TGMD-3 has demonstrated a high level of validity and reliability [21]. Other tests have adopted a similar structure, for example, in Australia the Get Skilled Get Active (GSGA) and the Victorian Fundamental Motor Skill Manual (Victorian FMS manual) [22, 23].

Note that the TGMD-3, GSGA, and other FMS assessments face concerns over their ecological validity due to their static nature, impacting real-world applicability and PE learning support [24–26]. They often assess isolated discrete skills [25]. Consequently, newer tests like the Canadian Agility and Movement Skill Assessment (CAMSA) [25] and Dragon Challenge (DC) [27] emerged, emphasizing a dynamic, ecologically-valid approach. These tests evaluate multiple movements consecutively and integrate sport skills and other aspects like coordination, balance and agility. Despite the strengths of both batteries, including the time it takes to complete the assessment, ease of scoring, and ability to assess in a group setting, assessing FMS in this capacity is not without limitations. Specifically, considering the continuous nature of the test, it is likely that the accumulating fatigue from successive tasks may negatively affect the performance of the final tasks and subsequently the overall test score. Furthermore, the complex consecutive sequences of skills performed, combined with time constraints, can compound stress and increase the likelihood of errors.

### Challenges and concerns in existing FMS evaluation

Although the development of FMS should be a public health priority, there remain many barriers preventing FMS from being properly developed in and out of PE classes. Baghurst et al. [28] examined how skill proficiency testing is conducted in PE teacher education (PETE) programs. They found that the majority of faculty members who worked within a collegiate PETE program believed that skill proficiency for PETE students was important, but only 46% of programs reported testing motor skills as part of their program, while 59% of programs tested physical fitness. They also reported a lack of uniform or consistent method to assess FMS and specific sport skills [28]. Other studies have noted barriers in the assessment of FMS in school settings including a belief of needing to enlist another teacher to help assess motor skills [29], increasing workload stress [29], deficits in validity and reliability of assessment tools [30], and focus on quality movement assessment [31]. PE teachers also often have to deal with large numbers of students in the classroom, limited class time and the fact that assessment may not be engaging nor enjoyable for students [32].

The evaluation of FMS proficiency for school-aged children is lacking in many countries, including Poland, and many countries have reported that the number of children that have mastered FMS is low [33–35]. In a study conducted in United States, De Meester et al. [35] found that almost 80% of children aged 6 to 11 years old presented low levels of FMS, while Brian and colleagues [36] cited that ~ 77% of preschool-age American children were considered at-risk for developmental delay (scored at or below the 25th percentile). Bolger et al. [33] reported that the global level of FMS of children aged 6–10 is ‘below average’ compared to normative data collected in 1997–1998. Rainer and Jarvis [37] showed that the overall FMS proficiency levels of Welsh children aged 10 to 11 years were low, with fewer than 10% of both boys and girls demonstrating complete mastery in any of the FMS. Similarly, research by O’Brien et al. [34] found that overall skill performance among Ireland adolescents aged 12 and 13 is low, highlighting the fact that almost 90% of students did not achieve mastery level in locomotor skills (e.g., running, skipping, jumping) or that only 11% of students in their study displayed advanced FMS proficiency. The FMS proficiency of Australian children aged 9–15 was also identified as low by the authors of a 13-yr report of motor competence, highlighting the fact that vertical jump performance significantly decreased from previous assessments [38]. Considering the low levels of FMS globally, it seems that more awareness-raising activities among policymakers, teachers and parents are needed.

### Rationale for a novel assessment tool

The decreasing level of FMS among students in many countries emphasizes the need to pay more attention to teaching and monitoring these skills during PE classes. However, assessing FMS can be challenging due to difficulties in objective assessment and inappropriate adjustment of test tasks to students’ movement-related needs. Furthermore, the decline in FMS proficiency is increasingly evident in adolescents, as indicated by previous studies [34, 38, 39]. Given these challenges, there is also an urgent call for refined assessment tools and tailored instructional methods to effectively address and enhance FMS proficiency in this critical developmental stage.

Cultural differences may influence which FMS are prioritized in different regions of the world, impacting the validity and reliability of FMS tests. For example, FMS tests developed in Western cultures may prioritize skills like throwing and catching, while tests developed in Asian cultures may prioritize balance and coordination. Notably, even within Western cultures, there are significant variations. Poland, a European country with a Central-Eastern cultural backdrop, offers a prime example of the necessity for a tailored evaluation tool. While it aligns with many Western norms, the overwhelming evidence pointing to the lack of FMS proficiency in this demographic accentuates the importance of developing a tool suited to this European context. The above rationale, combined with a desire to promote deliberate practice in teaching and learning FMS, served as the driving force behind the design and development of the new Fundamental Motor Skills in Sport (FUS) test. The purpose of this manuscript is to outline the development of the FUS evaluation tool and examine the initial psychometric evidence.

## Methods

**Fig. 1:**
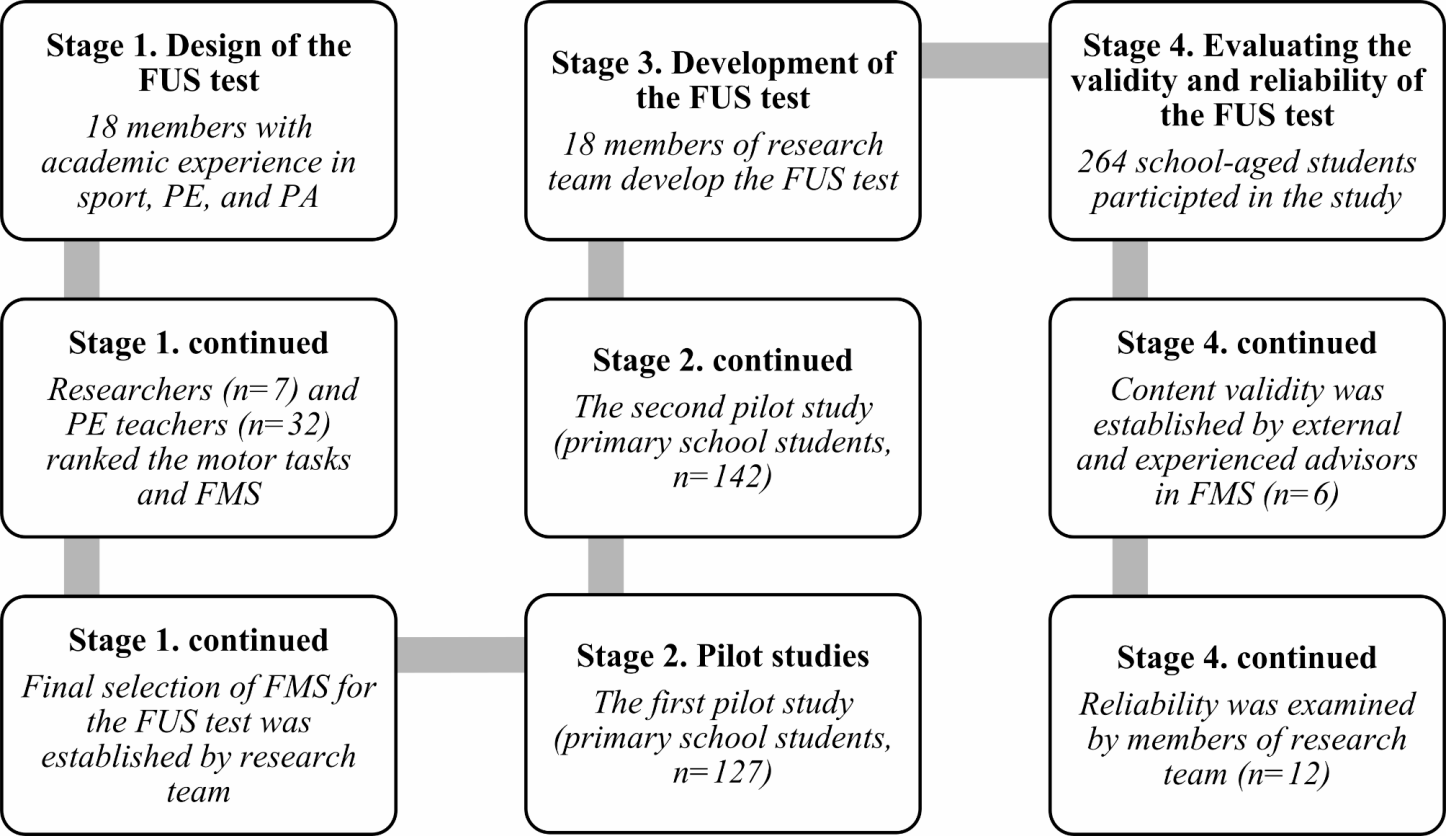
The study design

The present study was comprised of four stages (Fig. 1). The first included the design of a new FMS tool, the FUS test, by a panel of researchers. In the second stage, two pilot studies were conducted to examine the feasibility and acceptability of the proposed FUS assessment. The third stage involved the development of the FUS test. The last stage was focused on the examining the validity and reliability of the FUS. In accordance with the COSMIN taxonomy of measurement properties, content validity as well as several reliability measures were evaluated including: inter-rater, intra-rater, test-retest reliabilities and internal consistency were assessed [40]. The study design and testing protocol were approved by an institutional Research Ethics Committee.

### Stage 1. Design of the FUS test

The initial step was to establish a research team of 18 members with academic experience in sport, PE, and physical activity. Within the research team, seven members had specialized experience in motor development or motor learning research. Each of these members has authored or co-authored multiple peer-reviewed articles focused on the learning and development of motor skills; all have been conducting research in this field for at least seven years. The remaining members are researchers in sports, physical activity, and physiotherapy, all of which have experience teaching motor skills. Each team member conducts either theoretical or practical university classes related to motor skill learning or development.

A five-step, evidence-based practice (EBP) approach was employed by the research team to design the FUS test. This involved asking a question, finding evidence, evaluating the evidence, incorporating the evidence into practice, and reevaluating the evidence [41]. At the beginning (Step 1) the following question was formulated: How can we develop a culturally relevant, valid, and reliable assessment tool to evaluate FMS proficiency in children and adolescents, considering the unique needs and challenges of students in European regions like Poland? During this phase, the critical components of the research problem were identified, encompassing the population, intervention, comparison, and outcome (PICO).

Step 2 ‘Find Evidence’ entailed conducting a comprehensive literature review to gather evidence on existing FMS assessment tools. The best available evidence involved research, including original studies and systematic reviews. Research studies were eligible for inclusion if they focused on the validation of assessment tool measuring FMS, published in a peer-reviewed journal and analyzed a population consisting primarily of children and adolescents, specifically within the age range of 7–14 years. Studies not published as full articles were excluded from this review. Additionally, the research team gathered primary data by interviewing PE teachers (*n* = 32) and 9–14 year old students (*n* = 75) about their preferences and experiences concerning FMS and sports.

Upon gathering a pool of evidence, the team conducted a thorough analysis to identify common FMS relevant to the study population, and also analyzed the reliability and validity of existing FMS assessments (Step 3 ‘Evaluate Evidence’). Further, the research team identified 17 skills (e.g. running, jumping, catching, galloping, marching, climbing, swimming) and 20 motor tasks (e.g. jumping rope, jumping onto and off of a box, control a ball in a slalom dribble, cycling, throwing and catching a frisbee) common in sport and physical activity that represented the range of FMS proficiency of school-aged children (age range = 7–14 years). Subsequently seven researchers among the research team, who had experience with motor development or motor learning research, ranked these skills and tasks according to the best match they had with FMS definition (i.e., early movement behaviors that establish the foundation for later movement experiences [4]). From this shortened list that involved 9 skills and tasks, PE teachers then selected a set of skills and tasks most relevant to the goals of PE. The researchers and PE teachers also ranked the most important considerations associated with assessing FMS in school settings. The final six FMS were selected by considering information collected in a survey of the researchers, teachers, and students, in addition to three meetings held by a collective discussion group comprised of members of the research team. The final selection of skills for the FUS test was guided by the following criteria: (i) degree of sports utility that promote engagement in a broad range of physical actives, (ii) the fundamental nature of the skills to build a foundation for acquiring advanced skills, (iii) ensuring the comprehensiveness assessment, (iv) the ability to identify the components that are most important for skill mastery, and (v) ease of skill evaluation in an applied setting. The attributes considered in selecting the tasks included in the FUS test were: (i) the potential for an accurate skill assessment, (ii) a balanced trade-off between task complexity and simplicity, (iii) the feasibility of conducting the assessment in a school setting, (iv) ability to assess skills under dynamic conditions, (v) attractiveness of the task, (vi) the ability to assess several skills together while performing the task, (vii) ability to modify the task as needed.

### Stage 2. Pilot studies

After the initial development of the FUS test, the fourth step of the EBP approach ‘Incorporate the Evidence into Practice’ was applied. Two pilot studies were conducted to examine the feasibility and acceptability of the proposed test format. Two primary schools were randomly chosen from a total of nine in one Polish town, Biała Podlaska, for participation in this research. This selection aimed to ensure a diverse representation of students from different school settings. Following this, each of the participating schools provided a list of classes spanning all primary grades (from grade 1 to grade 8). To ensure balanced representation across the age range of 7–14 years, one class was randomly selected from these lists for each age group. For the first pilot study, a total of 127 children (61 girls and 66 boys) from the first school and 13 members of the research team participated after approval from the school headmaster, teachers, and parents. The number of students in each grade ranged from 12 to 17. One week later a second pilot study was conducted by 13 members of the research team which examined 142 children at the second randomly selected school. Again, the sample involved randomly selected classes from all primary school grades, including 66 girls and 76 boys. Class groups ranged 15 to 20 students.

The aim of the first pilot study was to assess the children’s ability to perform each task within the FUS and to verify if the research team could sufficiently evaluate all skill components. The research team then met and discussed concerns and differences based on their evaluations and made minor changes related to testing environment, equipment and task execution. Based on results of the first pilot study, it was determined that modifications needed to be made to four of the FUS tasks in order to minimize the influence of body build and physical abilities on FMS outcomes. For example, the modifications involved adjusting the height of the hurdles so they were age appropriate, changing the size of the balls, adjusting the distance and size of targets, and providing simplified rules for younger participants. The aim of the second pilot study was to validate the changes that were made to the assessment protocol following the initial pilot study. The changes made after the first pilot study proved valid. No new modifications were made after the second pilot study. Based on the results of the two pilot studies, the research team finalized the FUS test which is described in the following section.

### Stage 3. The development of the FUS test

Following the completion of the pilot studies the research team determined that the FUS test would include the assessment of six FMS: running over hurdles, jumping rope, forward roll, bouncing a ball, overhand ball throwing and catching, and kicking and stopping a ball. For each activity, the administrator assesses the level of mastery by evaluating key performance components of each task. Each task is assessed by 5 criteria which have been organized in a mixed process-oriented and product-oriented structure.

#### Hurdles

The task is to run over three hurdles (obstacles) in the 30 m run as fast as possible. The criteria in this task are as follows: ***criterion 1***. the run-up to the first hurdle is fast, knees are lifted high and elbows are bent; ***criterion 2***. there is no slow down prior to hurdle clearance, and there is clear forward movement during the take-off that precedes hurdle clearance; ***criterion 3***. body moves flat over the hurdle, the trunk leans forward, the trail leg moves quickly forward (without stopping); ***criterion 4***. stride pattern between the hurdles is rhythmic, the number of strides between particular hurdles is the same ***criterion 5***. there is no slow down after hurdle clearance, balance is maintained on landing and the run is continued in a straight line.

#### Jumping rope

The task is to perform rhythmic and continuous jumps over the rope for 10 s. The following criteria apply to this task: ***criterion 1***. jumps are performed continuously (without stopping); ***criterion 2***. jumps are rhythmic and single, with short ground contact time and landing on the ball of the feet; ***criterion 3***. arms are bent and held close to the trunk, and the rope is moved using the rotation of forearms and wrists; ***criterion 4***. knees and hips are slightly bent during flight and landing; ***criterion 5***. jumps are performed vertically with jumps initiating in the same designated area, with the trunk upright, feet parallel at a hip width apart.

#### Forward roll

The task is to perform a forward roll starting and ending in a squat position with hands on the ground. The following criteria are considered for this task: ***criterion 1***. the task is started in a squat position with both hands placed on the mat and the chin tucked into the chest; both legs are extended equally to push off the ground; ***criterion 2***. rolling over the back is performed without stopping and with the chin tucked; ***criterion 3***. symmetry of movement is maintained while rolling, legs are bent and tucked to the chest; ***criterion 4***. forward roll is performed in a straight line; ***criterion 5***. the task is completed in a squat position with hands placed on the ground in front of the toes.

#### Ball bouncing

The task is to bounce the ball while walking for 10 m and running an additional 10 m, for a total distance of 20 m. In this task, the criteria are: ***criterion 1***. in the first 10 m of the test the ball is rhythmically bounced at hip height with the top of the ball remaining below the chest while walking in a straight line; ***criterion 2***. the second 10 m of the test is covered running and bouncing the ball with the ball remaining relatively close to the body; ***criterion 3***. the whole distance (20 m) is covered bouncing the ball in front of and slightly to the side of the body. The ball is not carried during the duration of the test; ***criterion 4***. the elbow and wrist are extended when the ball is pushed toward the ground. The ball is controlled with the tips of the fingers; ***criterion 5***. the trunk is upright while the ball is bounced (students aged 7–9) or eyes are focused forward while the ball is bounced (students aged 10–14).

#### Throwing and catching

The task is to perform a one-handed overhead throw with a run-up, hit the targeted area of the wall with the ball, and then catch the ball with one or both hands after it bounces against the wall. The task must meet the following criteria: ***criterion 1***. the run-up is performed continuously without crossing the line marked on the floor; ***criterion 2***. the throw is initiated with the throwing arm is brought back and the foot of the opposite leg is clearly in front of the body; afterward, the overhead throw is performed; ***criterion 3.*** the ball hits the wall above the line (in the target area); ***criterion 4***. the ball is caught, and hands do not touch the chest; ***criterion 5***. the student remains behind the designated line when catching the ball.

#### Kicking and stopping a ball

The task is to direct the ball to the target area by kicking the ball with the foot and hitting the target area marked on the wall, and to stop the returning ball with the foot. The criteria in this task are as follows: ***criterion 1***. the run-up is performed continuously, and the line marked on the floor is not crossed following the kick; ***criterion 2***. the kicking leg is bent at the knee during the backswing for the kick, the non-kicking foot is placed beside the ball; ***criterion 3.*** the ball is kicked with the instep, top, or the side of the foot; ***criterion 4.*** the ball hits the target area marked on the wall, returns immediately to the student, and crosses the line of the designated area marked on the floor; ***criterion 5***. after hitting the target area, the ball is stopped with one foot in the designated area.

In terms of scoring, the participant is awarded “1” point for each criterion met and “0” points when the criterion is not met. Points are only given when criterion is clearly satisfied. Two attempts are performed for each prescribed task. The trial with the higher score is used for further analysis. Performances are video recorded and scoring is completed through an analysis of the video-recordings completed by assessor. Alternatively, live scoring based on performance of each task could be conducted immediately following the trial. If live scoring is conducted, it should be done by individuals who are experienced in assessing the components of skills that are evaluated in the FUS test. It is recommended that the assessor completes 8–10 h of training in the use of the FUS test prior to scoring this at the time of the assessment.

Similar to previous research [34, 42], four levels of mastery for each skill were established: ‘full mastery’, ‘near mastery’, ‘some mastery’, and ‘poor’. ‘Full mastery’ is achieved when all skill components are successfully performed (scored 5 points). ‘Near mastery’ is obtained when all but one component is performed correctly (scored 4 points). ‘Some mastery’ is accomplished when the execution of three components is correct (scored 3 points). If the performance of two or fewer components is properly executed, the level is considered ‘poor’.

Subsequently, the total of all six FUS skills provides the basis for evaluating overall FMS proficiency at four levels. ‘Excellent FMS proficiency’ is obtained when the student fully mastered all the assessed six FUS skills (scored 5 points for each skill) or mastered all but one skill that is ‘near mastery’ (4 points was scored). ‘Good FMS proficiency’ is reached when the student was at least ‘near mastery’ for each FUS skill (scored at least 4 points) and when the student did not meet the requirements established for ‘excellent FMS proficiency’. ‘Elementary FMS proficiency’ level is accomplished when the student scored at the ‘some mastery’ level for each assessed skill (scored at least 3 points) and when the student did not meet the requirements established for the ‘excellent FMS proficiency’ and ‘good FMS proficiency’ levels. The fourth level ‘insufficient FMS proficiency’ is achieved, when skill performance did not meet the requirements established for ‘excellent FMS proficiency’, ‘good FMS proficiency’ and ‘elementary FMS proficiency’ levels.

Prior to testing each skill, students are briefly told why this skill is important, how to perform the task and what skill components will be evaluated. Subsequently, students are provided verbal instructions and they are given a visual demonstration of the whole task by a trained administrator. Participants were provided standardized instructions designed to direct attention externally. According to the constrained action hypothesis, an external focus of attention supports motor learning and performance due to improvements in movement automization, resulting in more optimal performance compared to instructions which direct attention internally or neutrally. Studies have shown that using an external focus of attention is beneficial for throwing [43], catching [44] and jumping [45] in children, including children with Developmental Coordination Disorder [46]. It is worth noting that instructions that support the performance of the whole task also directly addresses at least one criterion in each task. For example, the instruction for the forward roll in the FUS promoted the adoption of an external focus of attention by instructing the participant to “perform a forward roll along the line. All participants perform one familiarization (i.e., practice) trial for each task followed by two formal trials. No verbal feedback on performance is given during and following each trial. All trials are recorded using a video camera or smartphone. The recording method, distance and camera angles are specified with each task to ensure consistency in data collection. For more information on the FUS testing procedure, please refer to the test instructions provided in the manual for teachers “Test of Fundamental Motor Skills in Sport” (Supplement 1).

### Stage 4. Evaluating the validity and reliability of the FUS test

In the fifth and final step of the EBP approach ‘Reevaluate the Evidence’, rigorous psychometric testing was conducted to establish the content validity, inter-rater reliability, intra-rater reliability, and test-retest reliability of the FUS test. This stage of study included 264 school-aged students in grades 1–3 (7–9 yrs; *n* = 81), 4–6 (10–12 yrs; *n* = 89) and 7–8 (13–14 yrs; *n* = 94), including 139 girls and 125 boys from six public schools randomly selected and stratified regarding place of dwelling (2 rural, 2 suburban and 2 urban schools) from a list of schools which participated in a nationwide project promoting extra-curricular sport activities. The project involved more than 100,000 school-aged children and 6,600 PE and early primary school teachers.

To establish content validity, six advisors, all with a research background in motor learning or development, out of the 12 invited to participate in the study completed an online questionnaire. Using a four-point Likert scale (1 = the item is not relevant to the measured domain; 2 = the item is somewhat relevant to the measured domain; 3 = the item is quite relevant to the measured domain; 4 = the item is highly relevant to the measured domain [47]), advisors rated the essential components for the selected FUS items. Means greater than 3.0 for each item were considered acceptable.

Before reliability testing, the research team underwent specific training related to the assessment. The 12 members of research team were divided into six pairs and each pair trained to evaluate one motor task. Each pair of evaluators were content experts in regards to the skill they were tasked to evaluate. Specifically, the hurdling assessment was evaluated by experts in track and field, jumping rope was assessed by experts in combat and strength sports, the forward roll was assessed by experts in gymnastics, bouncing the ball by experts in basketball, throwing and catching the ball by experts in volleyball and handball, and the kicking and stopping a ball was assessed by experts in football (soccer). Subsequently, using records from the pilot studies, each pair individually and together improved their expertise in evaluating the prescribed task per the FUS protocol. This process required approximately 15 h of their time.

Data collection occurred in May and June 2022 during regular PE classes. Six of the four-person teams (2 research team members and 2 postgraduates) administered and recorded the FUS test in the 6 participating schools. At the beginning of the lesson, participating children were divided into four groups of 3–6 students. All participants were assigned numerical codes to maintain anonymity and facilitate later video analysis. Two members of research group demonstrated, administered, and recorded two tasks: throwing and catching a ball along with kicking and stopping a ball, or jumping rope along with the forward roll. The remaining two tasks (ball bouncing and hurdles) tasks were demonstrated, administered and filmed by one researcher. Each class session took 45–50 min, including the introduction and warm-up. A range of 14 to 22 students from each PE class participated in a measurement session. Students performed two trials without any feedback after the familiarization trial, but general positive encouragement was given to all participants following the conclusion of each task. Testing sessions occurred indoors and outdoors. Each trial was videotaped and then evaluated by a pair of trained researchers. All tasks were recorded using one tripod-mounted video camera (Lamax W 9.1, Poland). The MP4 video format and 1920 × 1080 resolution were used in all recordings.

A total of 264 students were used to calculate descriptive data for FMS proficiency, in addition to determining internal consistency (Supplement 2). Inter-rater reliability was assessed by examining how consistent scores were from the two assessors in each pair when they scored trials from the same observed attempts. Each pair of assessors evaluated the performance of 212 students on a task (Supplement 3). The order in which the scored video footage was reviewed was randomized between examiners. To examine intra-rater reliability, researchers investigated the consistency of scores for 130 video recordings of student performances when they were reassessed after a four-week interval (Supplement 4). Test-retest reliability was carried out in two schools, involving 28 students. In both cases only one researcher conducted the measurements during PE classes; there was one week between the two assessments (Supplement 5).

### Statistical analyses

Descriptive statistics were reported using means and standard deviations. Validity of the FUS test was achieved by comparing the assessments of the six advisors with the use of content validity index (CVI), consistent with the protocol described by Polit et al. [48]. Specifically, CVI was calculated by dividing the rating number of ‘3’ or ‘4’ provided by experts by the total number of the experts. A CVI greater than or equal to 0.83 was considered acceptable. Inter-rater and intra-rater reliability assessments were conducted using statistics and two-way mixed-effects modeling, single measures absolute agreement, and intraclass correlation coefficients (ICC). Cohen’s kappa coefficients were interpreted according to the classification proposed by Landis and Koch [49], and the percentage of observed agreements was calculated. Additionally, 95% confidence intervals (CI) were calculated for both reliability measures: Cohen’s kappa and ICC. Pearson correlation was used to test relationships between variables, and the internal consistency of the FUS test was assessed using Cronbach’s alpha. Limits of agreement (LOA) defined as mean difference ± 1.96*SD of the difference were calculated. One-sample t-test was used to check for systematic bias, while Pearson correlation was calculated to estimate proportional bias. The test-retest reliability was additionally assessed using two-way mixed-effects modeling, single rater, absolute agreement ICCs. For significance testing, the level of statistical significance was set at alpha = 0.05. Data were analyzed using SPSS 27 for Windows (SPSS Inc., Chicago, USA).

## Results

### Content validity

Mean (± SD) scores and CVIs of critical features for the FUS test are presented in Table 1. All aspects of the FUS test were rated relatively high by advisors experienced in FMS (mean value > = 3.0), reaching maximum CVI values. The characteristics that pertain to the proper identification of the performance criteria by the FUS test were rated to be high on average, with the smallest dispersion of 3.83 ± 0.41, while representativeness of FMS proficiency, scoring procedures, and feasibility in a school settings demonstrated the lowest value (3.33 ± 0.52).

**Table 1:** Mean (± SD) ratings by advisors and content validity index (CVI) of the FUS test characteristics (*n* = 6)

	Mean ± SD	CVI
How well does the FUS test assess FMS proficiency in school-aged children and adolescent?	3.33 ± 0.52	1.00
Does the FUS test involve sport useful skills?	3.67 ± 0.52	1.00
Do the FUS tasks allow for the assessment of the FMS of children aged 7–14?	3.50 ± 0.55	1.00
Are the skill components and performance criteria for each skill well identified?	3.83 ± 0.41	1.00
Is the scoring procedure correct?	3.33 ± 0.52	1.00
Is the test feasible in a school settings?	3.33 ± 0.52	1.00
Does the test have the potential to support learning and teaching?	3.50 ± 0.55	1.00

### FMS proficiency scoring

The lowest scores were observed for the hurdling and jumping rope tasks, 1.37 and 2.97, respectively; while the highest scores were for kicking and stopping a ball (2.90 ± 1.30; Table 2). The average score obtained in each FMS task did not exceed 3 points. As a consequence, an overwhelming majority of students (92.4%) demonstrated an insufficient level of FMS proficiency, 6.1% of children showed elementary FMS proficiency and 1.5% good FMS proficiency, respectively (Table 3).

**Table 2:** Mean ± SD score of mastery of individual FMS in the FUS test tasks among students (*n* = 264)

Task (sub-test)	Score (mean ± SD)
Hurdles	1.37 ± 1.51
Jumping rope	1.37 ± 1.59
Forward roll	2.11 ± 1.49
Ball bouncing	2.24 ± 1.38
Throwing and catching	2.38 ± 1.20
Kicking and stopping a ball	2.97 ± 1.30

**Table 3:** Percentage and number of students representing consecutive levels of FMS proficiency (*n* = 264)

Level of FMS proficiency	Percentage of total students (%)	Number of students by sex (n)		Number of students by age range (n)		
		Boys	Girls	7–9 yrs	10–12 yrs	13–14 yrs
Excellent FMS proficiency	0	0	0	0	0	0
Good FMS proficiency	1.5	3	1	1	1	2
Elementary FMS proficiency	6.1	10	6	3	8	5
Insufficient FMS proficiency	92.4	112	132	77	80	87

### Reliability

For inter-rater reliability, Cohen’s kappa coefficients were greater than 0.75 showing substantial or almost perfect agreement between the raters and the percentage of observed agreements ranged from 80.2 to 89.6% for all FUS skills (Table 4). The high agreement between raters was confirmed by high ICC values (0.97–0.98), indicating excellent inter-rater reliability for all tasks utilized in the FUS test.

**Table 4:** Inter-rater reliability for skills assessed in FUS test tasks (*n* = 212)

Task	Percentage of observed agreements (%)	Cohen’s kappa (95% CI)	Agreement	ICC (95% CI)	Strength of ICC
Hurdles	84.0	0.78 (0.75–0.81)	Substantial	0.98 (0.97–0.98)	Excellent
Jumping rope	86.8	0.81 (0.78–0.84)	Almost perfect	0.98 (0.98–0.99)	Excellent
Forward roll	86.3	0.83 (0.80–0.86)	Almost perfect	0.98 (0.98–0.99)	Excellent
Ball bouncing	80.2	0.75 (0.72–0.79)	Substantial	0.98 (0.97–0.98)	Excellent
Throwing and catching	89.6	0.86 (0.84–0.89)	Almost perfect	0.97 (0.96–0.98)	Excellent
Kicking and stopping a ball	84.0	0.80 (0.76–0.83)	Substantial	0.97 (0.96–0.98)	Excellent

Similar to the results of the inter-rater assessment, the intra-rater reliability measures showed strong or almost perfect agreement of scores given by the assessors at both time points. On average, there was slightly lower reliability using Cohen’s kappa measures for the second time the rater scored the skill, especially for kicking and stopping a ball (Table 5). Nevertheless, all Cohen’s kappa coefficients exceeded 0.70, the percentage of observed agreements ranged from 84.6 to 93.1% and from 78.5 to 89.2% by each rater at time point 1 and 2, respectively. Excellent reliability was found for all tasks of the FUS test, indicated by high ICC values (> 0.96) and narrow 95% confidence intervals observed for both raters.

**Table 5:** Intra-rater reliability for skills assessed in FUS test tasks (*n* = 130)

Task	Percentage of observed agreements (%)	Cohen’s kappa (95% CI)	Agreement	ICC (95% CI)	Strength of ICC
Hurdles Rater 1 Rater 2	93.1 89.2	0.90 (0.84–0.96) 0.85 (0.77–0.92)	Almost perfect	0.99 (0.99–0.99) 0.98 (0.98–0.99)	Excellent
Jumping rope Rater 1 Rater 2	89.2 85.4	0.85 (0.78–0.92) 0.80 (0.72–0.88)	Almost perfect	0.99 (0.98–0.99) 0.99 (0.98–0.99)	Excellent
Forward roll Rater 1 Rater 2	90.0 89.2	0.88 (0.81–0.94) 0.87 (0.80–0.93)	Almost perfect	0.99 (0.98–0.99) 0.99 (0.98–0.99)	Excellent
Ball bouncing Rater 1 Rater 2	84.6 83.9	0.81 (0.73–0.88) 0.80 (0.72–0.88)	Almost perfect Substantial	0.98 (0.97–0.99) 0.98 (0.97–0.99)	Excellent
Throwing and catching Rater 1 Rater 2	89.9 86.2	0.83 (0.75–0.90) 0.81 (0.73–0.89)	Almost perfect	0.97 (0.96–0.98) 0.96 (0.95–0.97)	Excellent
Kicking and stopping a ball Rater 1 Rater 2	88.5 78.5	0.86 (0.79–0.92) 0.73 (0.64–0.82)	Almost perfect Substantial	0.98 (0.98–0.99) 0.97 (0.96–0.98)	Excellent

Several significant correlations were found between the result of individual tasks within the FUS test (Table 6). Ball bouncing showed a moderate, positive relationship with the forward roll (*r* = 0.34; p < 0.001) and throwing and catching a ball (*r* = 0.38; p < 0.001). Other correlations were low (*r* < 0.3) or not significant. Cronbach’s alpha value was relatively low (0.59).

**Table 6:** Internal consistency for skills assessed in the FUS test (*n* = 264)

Task	Hurdles	Jumping rope	Forward roll	Ball bouncing	Throwing and catching	Kicking and stopping a ball
Hurdles		0.19*	0.18	0.11	0.16	0.08
Jumping rope			0.18*	0.28*	0.21*	0.09*
Forward roll				0.34*	0.22*	0.10
Ball bouncing					0.38*	0.27*
Throwing and catching						0.19*
Kicking and stopping a ball						

* *p* < 0.05

The test-retest reliability measures of individual tasks in the FUS test are presented in Table 7. No significant systematic bias was observed as the mean differences of both scores were all close to zero (from − 0.04 to -0.1). About 82–96% of the differences fell within the 1.96 SD limits of agreement which were reflected less variability for kicking and stopping a ball task and slightly more variability for the forward roll. There was no significant correlation between the mean of both scores nor the differences of both scores for all tasks (*r* between 0.01 and 0.20; *p*-value between 0.30 and 0.96) indicating no proportional bias in the data. Moreover, calculated ICC values were comparably high for all tasks (0.95–0.97) and indicated excellent test-retest reliability.

**Table 7:** Test-retest for skills assessed in the FUS test (*n* = 28)

Task	Mean difference (SD)	Limits of agreement	% within limits of agreement	ICC (95% CI)	Strength of ICC
Hurdles	-0.04 (0.51)	-1.03–0.96	89.3%	0.97 (0.93–0.99)	Excellent
Jumping rope	-0.07 (0.60)	-1.26–1.11	96.4%	0.97 (0.94–0.99)	Excellent
Forward roll	-0,0.1 (0.63)	-1.34–1.13	96.4%	0.96 (0.91–0.98)	Excellent
Ball bouncing	-0.07 (0.54)	-1.13–0.99	89.3%	0.95 (0.90–0.98)	Excellent
Throwing and catching	-0.07 (0.6)	-1.26–1.11	96.4%	0.96 (0.91–0.98)	Excellent
Kicking and stopping a ball	-0.04 (0.43)	-0.88–0.80	82.1%	0.96 (0.92–0.98)	Excellent

## Discussion

### Overview of the study

This article discusses the development and evaluation of a new assessment tool for FMS proficiency in children and adolescents. The development process of the FUS test involved four stages, with the aim of creating a valid and reliable test to evaluate FMS proficiency. Stage 1 involved the establishment of a research team with motor learning and development, and sport experience background who aimed to design this test. Stage 2 consisted of two pilot studies that tested the feasibility and acceptability of the FUS test format. The studies were conducted on students aged 7–14 years, representing all primary school grades in Poland. Based on the results of the pilot studies, the research team finalized the FUS test. Stage 3 involved the development of the FUS test, which finally included of six FMS tasks: running over hurdles, jumping rope, forward roll, bouncing a ball, overhand ball throwing and catching, and kicking and stopping a ball. Each task is assessed by evaluating key performance components and criteria organized in a mixed process and product-oriented structure. Stage 4 was devoted to evaluating the validity and reliability of the FUS test. This stage involved 264 school-aged students from six public schools. Validity was established by a panel of six advisers with a background in motor learning or development, while reliability was assessed through internal consistency, inter-rater reliability, intra-rater reliability, and test-retest reliability. Overall, this study represents a comprehensive approach to the development and evaluation of an evidence-based and reliable assessment tool for FMS in children and adolescents. The FUS test has the potential to assist educators and coaches in the evaluation and improvement of FMS proficiency, which can positively impact children’s long-term health and well-being.

### The origin and rationale behind developing the FUS test

Nowadays children have fewer and fewer opportunities to develop FMS proficiency due to their sedentary behaviors [50]. Although PE class appears to be the ideal environment for improving FMS proficiency, the approach of physical educators frequently falls short of addressing the comprehensive public health objective of fostering physical competence, active lifestyles, and overall well-being in youth. This shortfall often arises from several challenges, such as a deficit in educators’ knowledge and training on FMS assessment [32, 51, 52]. Consequently, many school-aged students are under-skilled in FMS [3, 53], or their FMS deficits are not properly identified [32]. Decreasing levels of FMS proficiency are becoming more apparent in young age (12–13 years) and older aged adolescents [34, 38, 39], the situation is aggravated by the fact that there is still a lack of tools to assess FMS in a timely fashion within an adolescent aged population [54]. In Poland, there is a lack of systematic research and well-established tools for assessing FMS in school-aged children and adolescents. This lack of standardization and consistency in assessment makes it difficult to compare data across studies and to develop effective interventions to improve FMS proficiency. Therefore, we have developed a novel tool that is expected to provide a standardized and reliable method for assessing FMS in school-aged children and adolescents. While the FUS test was tailored to cultural and environmental factors specific to Poland, it is not limited to the Polish context and can be used in other countries due to its focus on skills necessary for participation in sports and physical activities worldwide. We assume that this cross-cultural applicability makes this test a valuable contribution to the international community, as it allows for standardized assessment and comparison of FMS across countries and cultures.

### The FUS test tasks and skills

The FUS test allows for the evaluation of FMS in school-aged students by assessing their proficiency in six different motor tasks related to sports: hurdles, jumping rope, forward roll, ball bouncing, throwing and catching, and kicking and stopping a ball. The first task of the FUS, running over hurdles, assesses proficiency in the combination of high speed running, unilateral jumping (i.e., leaping), rhythm and dynamic balance. The ability to run is a fundamental action which needs to be learned by all children [55]. Running requires coordination between the legs, arms, and torso. Running is essential for successful participation in many team and individual sports. It is also a critical skill for successful participation in many physical activity based games for children. An inefficient running movement pattern can contribute to early fatigue, and thereby limit opportunities to develop other FMS [56]. Running over hurdles can be intimidating for some children, especially if they are not used to this type of activity. Through successfully clearing hurdles, children can overcome their fears and build confidence in their capacity to manage physically demanding situations. Running over hurdles is a skill that requires a certain level of coordination, rhythm, balance and agility, which are important abilities in many playground games and effective locomotion. A consistent running rhythm helps to maintain a consistent stride length and frequency, which in turn helps to optimize running efficiency [57] and reduce the risk of injury. Note that mature movement patterns are rhythmically stable [58], conversely arrhythmic movements lead to asymmetry and depressed running performance. MacPerson et al. [58] suggest that the role of rhythm in motor learning and performance is essential and exercises aimed at maintaining a consistent rhythm could be considered a prophylactic against potentially disruptive cognitions and emotional states that inhibit movement fluency. Also, from a physiological point of view, rhythm leads to better running performance [59].

Rhythm is also an important component of the second FUS task, jumping rope, which assesses bilateral jumping ability, postural control, landing, and coordination of the arms and legs. Jumping rope usually involves consecutive bilateral vertical jumps while turning the rope around the body [60]. Because rebounds are quick, with very little bending of the foot, knee, and hip joints, it is considered a plyometric task that uses a specific movement pattern that supports jumping ability, power, and speed development [61]. During this task, the ability to re-establish balance using postural control mechanisms, through whole-body coordination, as well as kinesthetic awareness as a result of jumping repeatedly in the same spot are required for successful performance [62, 63]. Jumping rope also allows for the evaluation of neuromuscular control during landing, which is an important skill to learn to reduce ground reaction force injuries when playing sports such as volleyball, basketball, handball and gymnastics [64]. Jumping rope could be considered as a task which promotes neural plasticity through the modification of existing locomotor neural networks [65]. It is also worth noting that jumping rope has been shown to improve health-related outcomes and well-being in obese and non-obese children [63, 66–68], and jumping rope is a popular activity among children.

The third task of the FUS test is the forward roll. This task involves rolling across the back in a tucked position by starting in a squat position and ending in the same squat position. This skill is commonly used in gymnastics and combat sports (e.g. judo, wrestling). In other sports and physical activities when the risk of falling or losing balance is high (e.g. riding a bicycle or playing sports such as rugby, handball, soccer, athletics), mastering how to absorb ground impact while maintaining dynamic postural control when rolling may have beneficial effects for injury prevention. Note also that the forward roll stimulates the vestibular system which is responsible for maintaining balance, receiving information related to gravitational force, maintaining proper muscle tone, maintaining constant visual field during head movements as well as movement planning [69, 70].

The another task used in the FUS test assesses object control skills. Bouncing a ball (i.e., dribbling) is necessary to successfully play the games of basketball and handball. The ability to manipulate an object with your arms during locomotion movements is also important in other sports such as rugby, American football, tennis, and volleyball. Dynamic ball bouncing effectiveness depends on eye-hand coordination, dynamic balance, rhythmicity and coupling of movements [71]. The execution of this complex skill may provide information regarding the ability to synchronize movements along with visually tracking and physically manipulating a ball while performing a locomotor task.

The fifth task of the FUS test involves throwing and catching a ball, which is a necessary skill in many sports, recreational, and physical activity based games. The overhand throwing pattern is utilized in sports such as baseball, cricket, handball, American football, javelin, and throwing a ball for distance. Research has demonstrated that there is a similar overarm movement pattern of throwing and striking when performing a handball throw, volleyball spike, and tennis serve [72]. The variant of throwing used in the FUS test allows the test administrator to evaluate the coupling of movements performed during the approach run and throwing phase, arm range of motion, and throwing accuracy. Similar to throwing, catching is a skill that plays a key role in basketball, handball, baseball, rugby, and American football [73]. This skill is a fundamental action in ball sports and games because it requires anticipation, coordinated body movements as well as focus of attention [74]. Combining throwing and catching in the FUS test provides the possibility of recognizing whole-body coordination, kinesthetic, and spatial-visual abilities.

The last skill, kicking and stopping (i.e., trapping) a rolling ball are also skills adopted from many popular sports such as football (soccer), American football, and rugby. Similar to throwing and catching, whole-body coordination, kinesthetic and spatial-visual abilities may be examined in situations where the legs are extensively engaged [74]. Kicking and stopping a ball also provides an opportunity to evaluate coordination between the visual system and the lower extremities.

The FUS test, meticulously designed around six core motor tasks, offers a holistic approach to evaluating FMS in school-aged students. From the coordination required in running over hurdles, the rhythm in jumping rope, the balance in forward rolls, to the object control in ball activities, each task encapsulates a unique facet of coordinated movement patterns. Collectively, these tasks foster the development of an extensive skill set crucial for a spectrum of sports and routine activities, ensuring an individual’s readiness for lifelong physical engagement. In essence, the FUS test doesn’t merely gauge proficiency; it also sets the stage for a lifetime of physical agility and active participation in both recreational sports and daily tasks.

### Assessment and skill mastering criteria of the FUS test

The FUS test was developed to assess FMS proficiency, thereby understanding potential needs for future instruction, which may facilitate active and persistent participation in sports and physical activity. In line with the idea that assessments can be used to help instructors with specific aspects of movement performance [74], we qualitatively assessed FMS by adopting process-oriented criteria in each task. The qualitative assessment provides the teacher or skill assessor essential information for assisting the learner, indicating which components of skill have been mastered and which need additional practice. During the pilot studies, key components for effective performance of each task were identified. These task components refers to the actions of the whole body, or the movements of the arms, legs, trunk or performance outcome (e.g., criterion 3 in jumping rope task is met when “arms are bent and held close to the trunk, and the rope is moved using the rotation of forearms and wrists”, or criterion 4 is met when “knees and hips are slightly bent during flight and landing).

The FUS assessment uses a structured evaluation system to assess proficiency in FMS. The evaluation process for each skill in the assessment begins by assessing five performance criteria (components). Each FMS skill examined in the FUS test are graded on a 4-degree scale, ranging from ‘full mastery’ when all components are performed correctly to a ‘poor’ level when no more than two skill components are executed accurately. The level of mastery of each FUS test skill determines the level of overall FMS proficiency. The FUS test assigns four levels of FMS proficiency. For example, a student reaches the level of ‘good FMS proficiency’ when he or she scored at least 4 points in each FUS test task (item). However, when 3 points are scored in each item, the student achieves the level of ‘elementary FMS proficiency.’ Our findings show that none of the students reached the excellent level of FMS proficiency in the FUS test and only 1.5% students had ‘good FMS proficiency’. This not only underscores potential deficits in teaching and learning FMS for these students but also challenges the widespread misconception that FMS will naturally develop without external intervention. Furthermore, our findings suggest the absence of a ceiling effect in the FUS test. This contrasts with reported ceiling effects in previous FMS tests [75], a significant observation, especially concerning adolescents. We predict that a floor effect may not be present in the FUS test because the evaluated tasks are relatively easy to master with sufficient teaching, practice, and learning. However, this hypothesis should be tested and validated through further research and experimentation with a larger and more diverse sample population. In addition, we have introduced an ‘elementary FMS proficiency’ level in the FUS test evaluation system to support deliberate practice. This level is intended to aid in the development of FMS and motivate students to improve. By breaking down the assessment into smaller, more manageable steps, teachers can help students feel a sense of accomplishment and build a solid foundation for future success [11, 75].

### Validation of the FUS test

The impetus for designing the test was the assumption that the FUS test should be based on common activities found in various sports with the goal of increasing successful and persistent participation in a range of physical activities among school-aged children. Hence, the FUS test represents a broad repertoire of FMS which are directly and indirectly associated with a range of popular physical activities and sports worldwide, including games with balls, track and field or common playground games [75, 76]. Our findings support the conclusion that the FUS test represents independent skills, with each task representing an independent component of overall FMS proficiency. Further analysis showed that the skills used in the FUS test were found to have excellent content validity. Experts have consistently awarded high ratings across all the test characteristics, emphasizing its robust capacity to evaluate FMS proficiency. A standout feature of the FUS test is its emphasis on skills useful in sports, suggesting that these skills are not only foundational but also carry direct relevance to various sports. The versatility of the FUS test is evident in its suitability to assess FMS in a broad age range (7–14 years). The test is also commendably precise in delineating skill components and performance criteria via process-oriented criteria that may assist in teaching these activities. Other positive aspects highlighted include the scoring procedure, adaptability within school environments, and the potential it offers to bolster both teaching and learning processes. The FUS makes a unique contribution to the existing FMS literature by offering new assessments for sport specific skills which have not been captured in previous FMS assessment systems.

### Reliability of the FUS test

In terms of reliably assessing FMS, utilizing criteria which are too general or too specific could increase the potential disagreement between assessors or during consecutive measurements, therefore a compromise between general and specific evaluation approaches were adopted. The excellent inter-rater and intra-rater reliability of the FUS tasks confirmed the desired effect of the assessment was achieved. The reliability of the skill scores between test-retest was excellent, indicating that there was no significant learning effect. Therefore, the assessment is considered appropriate for monitoring performance over time. To minimize the influence of previous assessment attempts, it is recommended to have a minimum test-retest interval of one week. While the results of this study suggest that the FUS test is a reliable assessment tool for FMS, it’s important to note that the sample size used in the study was relatively small and limited to a specific geographic area, without considering the breakdown by gender, age and socio-cultural aspects [77]. To confirm the generalizability of these findings to other populations, further research involving larger and more diverse samples, which are split by gender and age, is needed. Nonetheless, the current study provides promising evidence that the FUS test is a reliable assessment tool that could be useful for monitoring the development of FMS in school-aged children and adolescents. It is important to note that the assessors in the current study were either coaches or researchers with expertise in sports. This could potentially restrict the generalizability of the results to populations of PE teachers, who are the intended audience for this test. PE teachers may have differing levels of experience and knowledge in assessing FMS [78], as compared to sports researchers and coaches. Hence, it is recommended that future studies verify the reliability of FMS assessment among PE teachers.

### The FUS test feasibility

The FUS test was developed as a tool to assess FMS proficiency for both researchers and PE teachers. The findings of our investigation provides preliminary evidence that the FUS assessment is a valid and reliable tool for assessing FMS, and it is also feasible to administer the test in a school setting during a PE class period. The researchers reported that the administration of the whole test, with video recording, including warm-up (5–6 total min), demonstrations of tasks by a teacher (average 1 min per task = 6 total min), familiarization trials (average 3–4 min per task = 22 total min), transitioning/ moving to the next task (1 min per task = 6 total min) and performance of two trials (30–50 s per task x 6 = about 55 min for 13 students and about 75 min for 15 students) involved 94 total minutes for 13 students (13 years of age) and 114 total minutes for 15 students (range 8–11 years of age). The overall estimated time to assess one student is 12–14 min (test administration = 7–8 min and evaluation = 5–6 min per student). This assessment time is longer than previous tests like the CAMSA (30–32 min for 20 students) [25] or DC (10 min per student) [27], but shorter than the TGMD-3 (15–20 min per student) [79]. Future research should assess the time needed to administer the FUS test by PE teachers and classroom teachers.

It should be noted that the assessment protocol and task performance procedures of the FUS test require one person, while in CAMSA and DC require two examiners or the contribution of another person (i.e., teacher or student) in some tasks (e.g., catching) is needed (TGMD-3, GSGA and Victorian FMS manual [22, 23, 79]). Note also that each task of the FUS test could be administered independently and individually as isolated assessments, therefore the teacher has the flexibility to conduct the test by adjusting the time frame to fit the curriculum and school settings. For example, one task evaluated in the FUS could be incorporated in one lesson within a larger PE unit. In such an arrangement, the part of the lesson preceding the performance of a given task should be standardized to ensure similar conditions in future measurements of that task.

### Strengths and limitations of the FUS test

A major strength of the FUS test is its assessment of FMS that are essential for a wide range of physical activities and sports, which makes it a comprehensive evaluation of motor function. The FUS test is also relatively easy to administer and score, requiring minimal equipment and training. Additionally, the FUS test has the potential to identify deficits in motor skill development early on, allowing for timely intervention and support to promote optimal motor function. The use of standardized tasks in the FUS test also allows for comparison of results across different populations and settings. Finally, the FUS test has the potential to be a useful tool in promoting physical activity and healthy lifestyles among school-aged children, as it can help identify areas of weakness and provide targeted interventions to improve motor skill development.

One limitation of the FUS test is that it is a performance-based assessment that requires a certain level of physical ability and understanding of the tasks being evaluated. This means that children with significant physical or cognitive impairments may not be able to complete the test accurately, and their motor skill development may not be fully captured. The FUS test was developed to provide a valid, reliable and feasible assessment of FMS proficiency for a wide range of school-aged students. Using video to score participants taking the FUS test is recommended, which could be considered a limitation of this assessment tool as the test administer may not have easy access to a recording device or the space in which to appropriately video the tasks. However, the authors of this test believe that new technologies should be used in PE lessons to increase objectivity and provide valuable information for teachers assessing FMS. Due to the growing possibilities for using smartphone applications for movement assessments [80], we can see the potential to develop a mobile application to administer FMS tests, such as work by Copetti and colleagues [81] who created a mobile application to provide pedagogical support for FMS assessment using the TGMD-3. There are of course other limitations to the present study that were addressed by Barnett et al. in their studies [75]. For example, more investigation is needed to further validate and test the repeatability of the findings we have reported. Additionally, future studies should test the efficacy of the FUS across larger and more diverse samples, appraise subgroup differences, explore sex effects, determine if the test discriminates for particular disabilities, and if the FUS test has predictive capabilities regarding talent identification. The findings and limitations of this study highlight the need for further research to optimize FMS assessment in children and adolescents.

## Conclusion

The importance of FMS in fostering confident, skillful, and enjoyable participation in physical activities throughout life cannot be overstated. With a concerning global decline in FMS proficiency among children and adolescents, there is an imperative need for systematic teaching, learning, and assessment in PE settings. Addressing this need, our study introduced the FUS test, specifically tailored to evaluate sport skill-based tasks, encompassing hurdling, jumping rope, forward roll, ball bouncing, ball throwing and catching, and ball kicking and stopping. Validated among a diverse age group of Polish students, the FUS test showed excellent content validity and inter-rater, intra-rater, and test-retest reliabilities. Moreover, its feasibility in school environments makes it a promising tool for standardized FMS measurement. By facilitating deliberate practice and providing a structured assessment, the FUS test offers a practical solution to the urgent need to enhance FMS proficiency in school-aged students.

## Future scope and practical implications

Although the target group for this test is school-aged students, this tool could be used successfully in sport preparatory schools and sports clubs to monitor FMS proficiency; however further reliability and validity work is needed with these subgroups. Early diversification approach or practicing different sports during the sampling stage (from 6 to 12–13 years) is believed to be beneficial for sport development due to the exposure to a variety of environmental, task, and psychosocial constraints, enhancing the motor, cognitive and perceptual skills needed for future successful sports specialization [82]. In a sport development context, it is worth noting that the three tasks utilizing balls in the FUS test allow for the comparison of the dominant and non-dominant side of the body and evaluate movement asymmetry. The ability to perform skills using both sides of the body is a desirable feature for many sports [83].

The FUS test has the potential to serve as a preliminary screening tool to identify the risk of coordination disorders among school-aged children. Deficits in fundamental movement skills (FMS) have been linked to disabilities such as DCD [84], therefore future work on the clinical validity of this assessment could examine whether the FUS test may be used to identify potential DCD risk. For instance, a score of 1 or lower for each skill on the FUS test may indicate an increased risk for DCD. DCD is estimated to affect 5–6% of school-aged children [85].

In conclusion, while the FUS test demonstrated promising results in its evaluation, further studies with varied groups of participants should be conducted to determine its applicability and reliability in different contexts.

### Electronic supplementary material

Below is the link to the electronic supplementary material.

Supplementary Material 1

Supplementary Material 2

Supplementary Material 3

Supplementary Material 4

Supplementary Material 5

## Data Availability

All data generated or analyzed during this study are included in this published article and its supplementary information files.

## Abbreviations

CAMSA: Canadian Agility and Movement Skill Assessment
CI: Confidence intervals
COSMIN: Consensus-based standards for the selection of health measurement instruments
CVI: Content validity index
DC: Dragon Challenge test
DCD: Developmental coordination disorder
FMS: Fundamental motor or movement skills
FUS: The Fundamental Motor Skills in Sport test
GSGA: Australia the Get Skilled Get Active
ICC: Intraclass correlation coefficients
LOA: Limits of agreement
PA: Physical activity
PE: Physical education
PETE: Physical education teacher education
SHAPE: Society of Health and Physical Educators
TGMD: The Test of Gross Motor Development
Victorian FMS manual: The Victorian Fundamental Motor Skill Manual
