# Supplementary material for: The fus test: a promising tool for evaluating fundamental motor skills in children and adolescents
Source: BMC Public Health. 2023 Oct 3;23:1912. doi: 10.1186/s12889-023-16843-w (PMC10548572; doi:10.1186/s12889-023-16843-w)
Supplement: Supplementary file 1 — Supplementary Material 1 [file 12889_2023_16843_MOESM1_ESM.pdf]

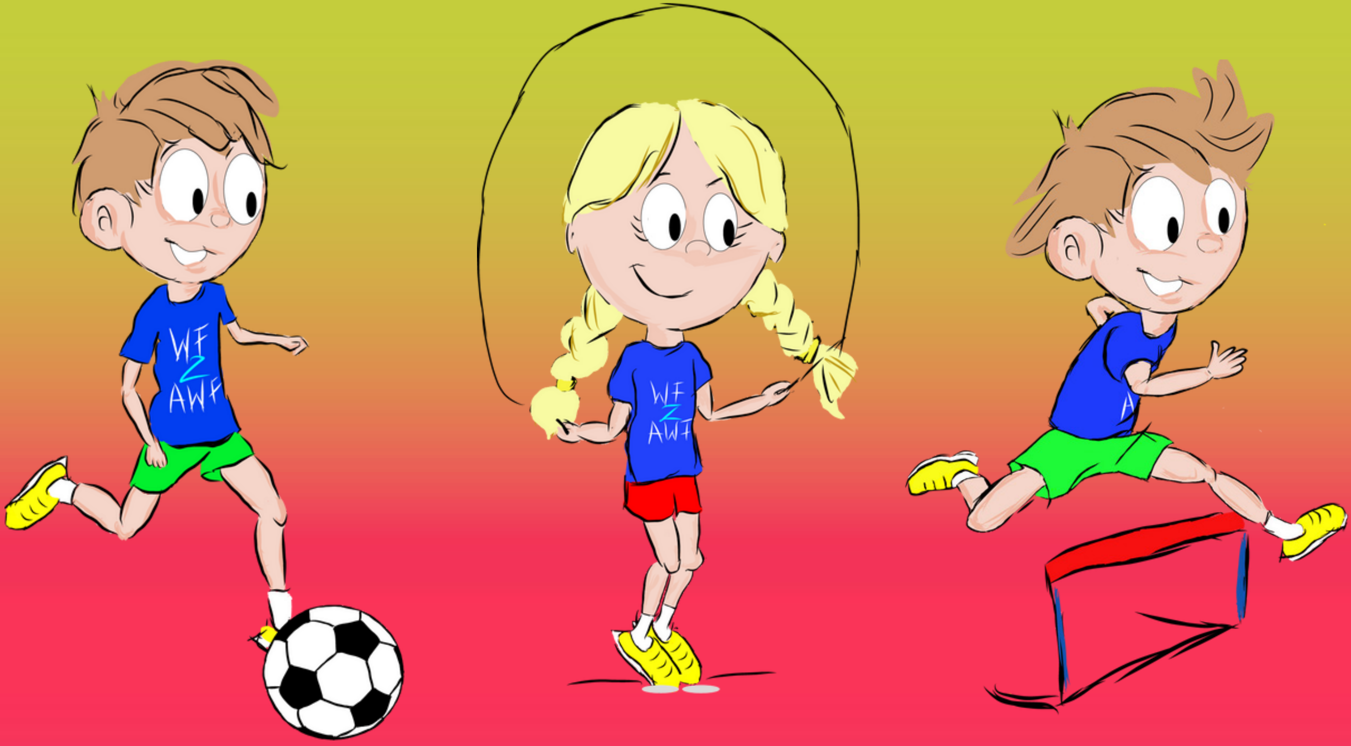

TEST OF FUNDAMENTAL MOTOR SKILLS

IN SPORT

FUS

A MANUAL FOR TEACHERS

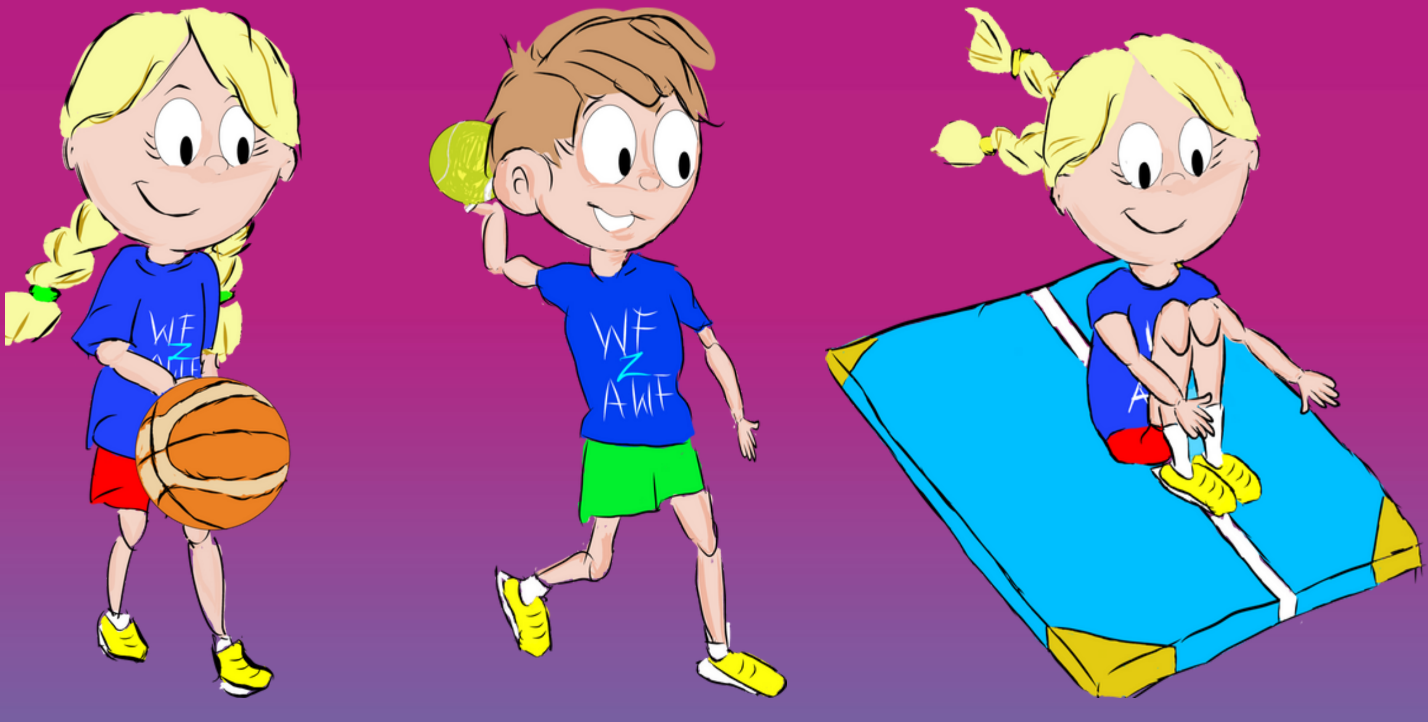

Active today,
healthy and skillful tomorrow!

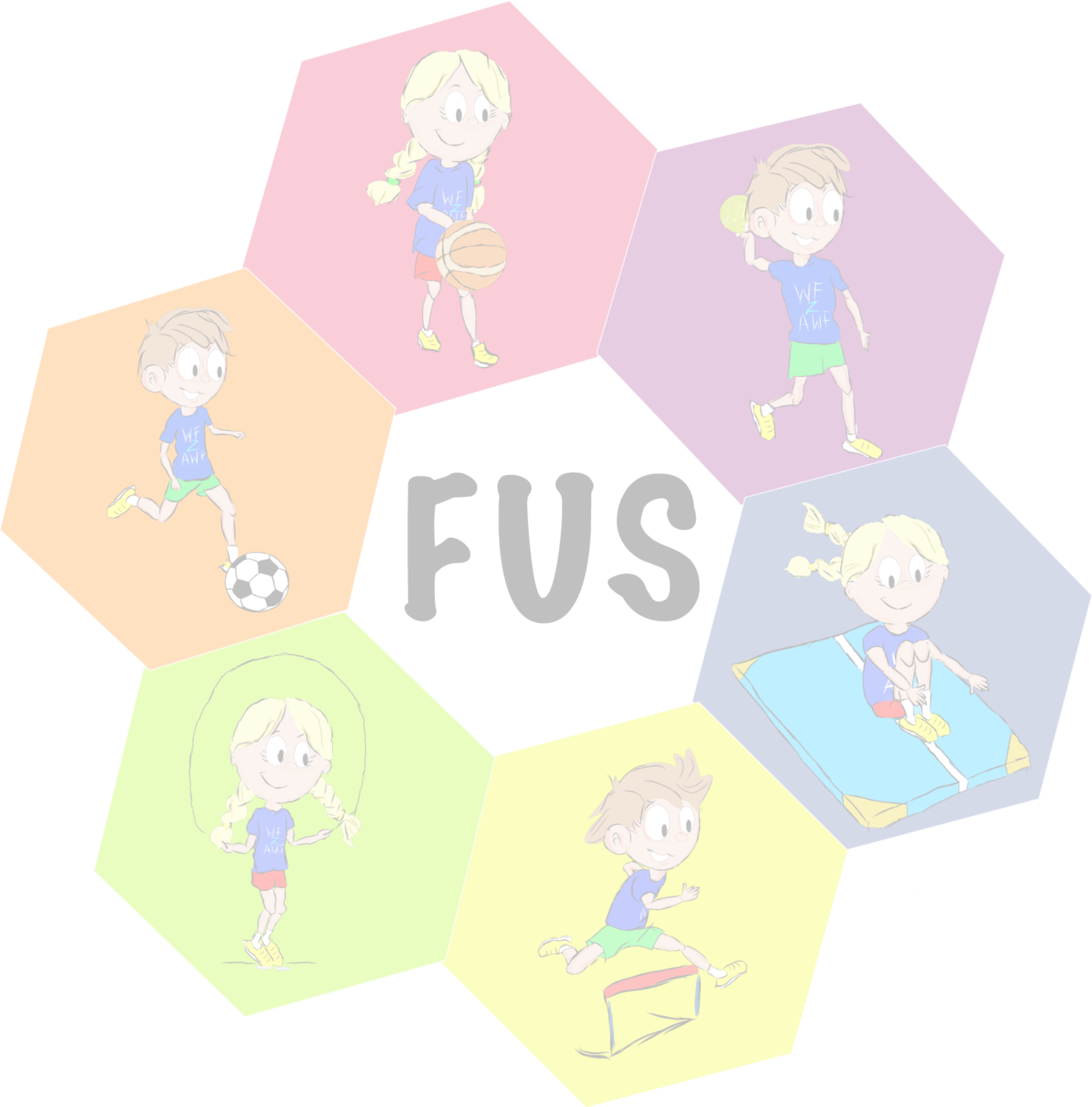

Józef Piłsudski University of Physical Education in Warsaw
Faculty of Physical Education and Health

TEST OF FUNDAMENTAL MOTOR SKILLS IN SPORT

FUS

A MANUAL FOR TEACHERS

Editor
Hubert Makaruk

Contributors
Hubert Makaruk, Jared Porter, E. Kipling Webster, Anna Bodasińska, Beata Makaruk, Janusz Zieliński, Marta Nogal, Paulina Szyszka, Michał Banaś, Michał Biegajło, Agata Chaliburda, Dariusz Gierczuk, Marcin Starzak, Bogusz Suchecki, Marcin Śliwa, Anna Kudelska, Bartosz Molik, Jerzy Sadowski

Citation: Makaruk H. et al. (2023) Test of Fundamental Motor Skills in Sport (FUS). A manual for teachers. Published by Józef Piłsudski University of Physical Education in Warsaw, Faculty of Physical Education and Health

Biała Podlaska 2023

Funded by the Ministry of Education and Science
'WF with AWF – Active Return to School after the Pandemic' programme
Agreement No. MEiN/2023/DPI/156

Ministry of Education and Science
Republic of Poland

Copyright © 2023 by Józef Piłsudski University of Physical Education in Warsaw
Faculty of Physical Education and Health

ISBN
978-83-61509-75-2

Editorial office
Józef Piłsudski University of Physical Education in Warsaw,
Faculty of Physical Education and Health in Biała Podlaska
Akademicka 2, 21-500 Biała Podlaska, Poland

Cover design and illustrations
Paulina Szyszka

Typesetting
Robert Wilczewski

Druk
Mazowieckie Centrum Poligrafii
www.drukksiazek.pl

Contents

Role of motor competence and fundamental motor skills in developing healthy lifestyle 5
Fundamental motor (sports) skills 5
Motor skill and its components 7
Stages of motor skill acquisition 8
Biological and social determinants of teaching and learning fundamental motor skills 8
Teaching and learning fundamental motor skills 10
Assessment of fundamental motor skills 12
Test of Fundamental Motor Skills in Sport (FUS) 13
FUS test guidelines 14
References 17
Hurdles 19
Jumping rope 23
Forward roll 27
Ball bouncing 31
Throwing and catching 35
Kicking and stopping a ball 39
Hurdles (shortened criteria sheet) 43
Jumping rope (shortened criteria sheet) 44
Forward roll (shortened criteria sheet) 45
Ball bouncing (shortened criteria sheet) 46
Throwing and catching (shortened criteria sheet) 47
Kicking and stopping a ball (shortened criteria sheet) 48

„Life is movement,
it is its very essence!"

A. Schopenhauer

Role of motor competence and fundamental motor skills in developing healthy lifestyle

One of the main goals of physical education (PE) is to develop healthy and physically literate individuals who are able to enjoy the benefits of lifelong physical activity. With this in mind, physical education curricula (and PE classes in particular) should stress the importance of motor competence improvement resulting in the acquisition of motor skills that are fundamental for the performance of a variety of physical activities [1].

Research results clearly indicate that children who have acquired fundamental motor skills are more willing to participate in physical activity compared to children who have not mastered such skills. Note that children who have not acquired skills as well as their peers may be excluded from participating in games and spend less time with peers. Moreover, due to such negative experiences, they may be unwilling to engage in physical activity in the future [2-4].

In summary, high levels of fundamental motor skills exert a positive influence on physical fitness [5], thus determining lifestyle and involvement in physical activity [3, 4, 6]. One may also presume that higher levels of fundamental motor skills contribute to better academic performance [7] and positively correlate with mental wellbeing [8].

Fundamental motor (sports) skills

Fundamental motor skills are basic and indispensable skills which determine participation in physical activity, sport and activities of daily living (e.g., walking safely down stairs). The most common skills that we use in a wide variety of sports include running, jumping, throwing, catching, bouncing a ball, and kicking.

Sport constitutes an excellent platform for developing fundamental motor skills [9]. Through sport, fundamental motor skills become sports skills, thus forming the ground for more specific and advanced skills used in a given sport [10].

When children manage to learn to run, jump, throw, catch, hit or dribble a ball during PE classes, the door to an active lifestyle is wide open and lessons themselves become a pleasure!

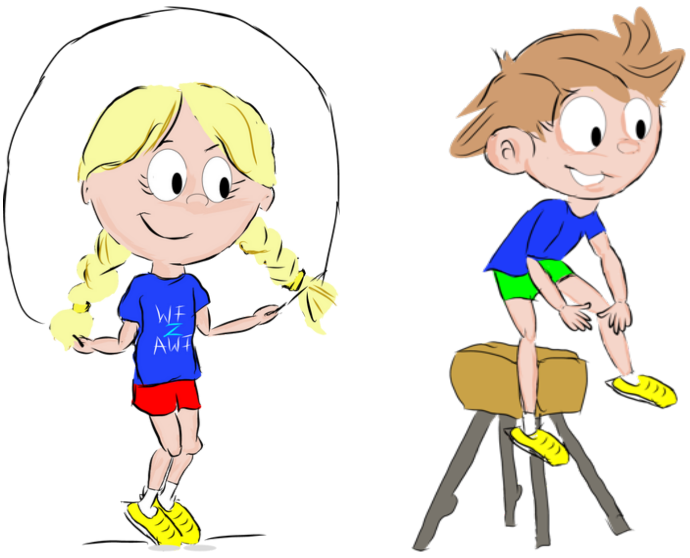

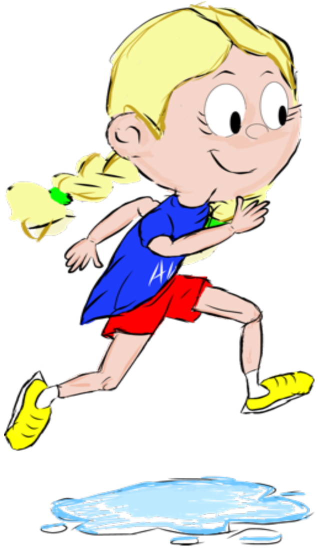

IF I CAN JUMP, I CAN ...

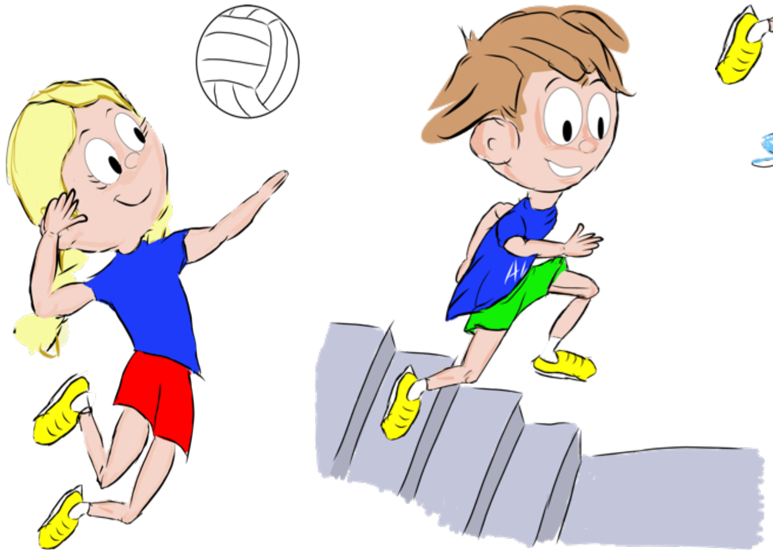

Motor skill and its components

Key components of motor skills were distinguish into movement structures that determine the effectiveness of performing an entire motor task. The acquisition of particular components of a motor skill often occurs at a different pace, and it depends on the age and physical fitness of a student. By knowing the basic motor components of a given skill, teachers can provide their students with precise instructions and feedback that facilitate skill acquisition. Furthermore, they can evaluate their students' progress in a valid and reliable manner. For example, the following components determine the effectiveness of kicking a football (soccer) ball: planting the non-kicking leg, controlling the range and speed of the kicking leg during the backswing for the kick, striking the ball and follow through of the kicking leg after contacting the ball.

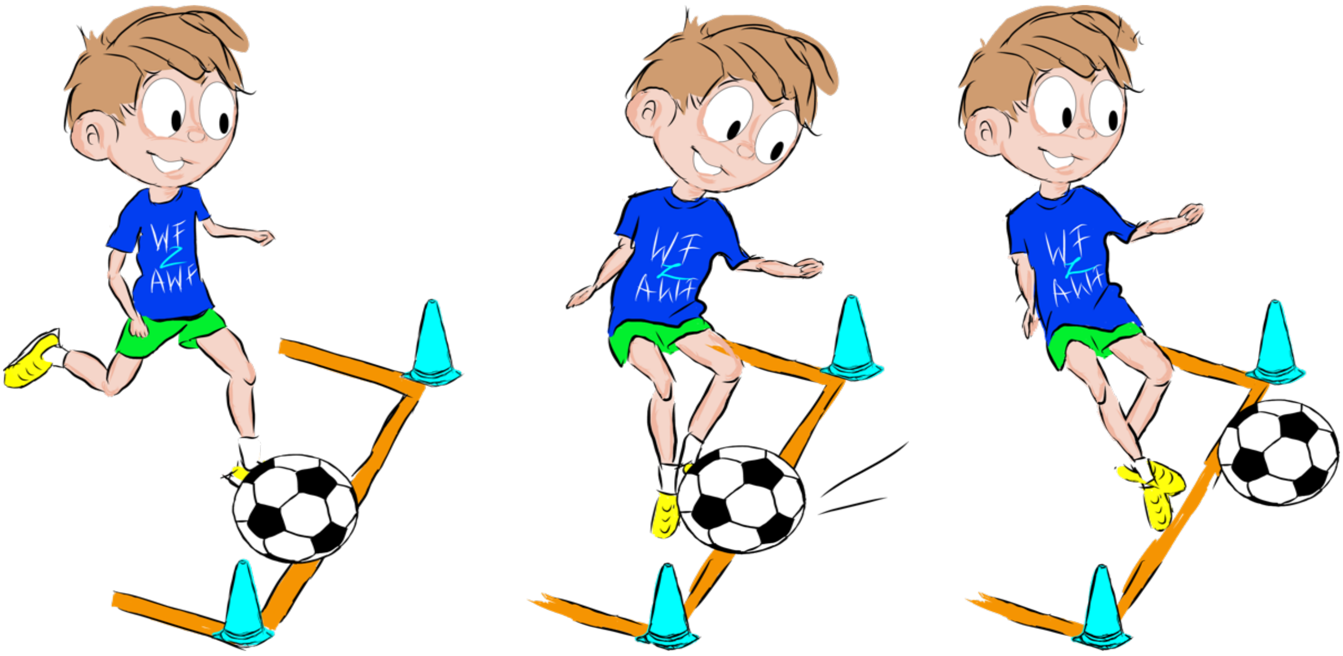

EACH MOTOR SKILL CONSISTS OF KEY COMPONENTS

Stages of motor skill acquisition

To put it simply, motor skill acquisition takes place in three stages. At the first (cognitive) stage, the learner should do their best to understand the nature and manner of performing a given motor skill. Here, putting different movement components together may challenging for the low skilled child. The first attempts are usually riddled with errors, and there is much variability across performance attempts. In addition, the moves are stiff and not coordinated. Feedback provided by the teacher in the form of instructions or demonstrations plays a pivotal role in the learning process. Stage two (associative) involves attempting to combine movement components into one smooth task performance. The learner is aware of fundamental errors committed during task performance. Feedback from a teacher still plays a key role at this stage; however, it is more precise as to guide the learner to a more correct movement. The third and final stage of learning (autonomous) is characterized by complete smoothness of movement reflected in high automaticity of task performance. Movements connected with a given skill become highly repetitive. Intrinsic (or sensory) feedback begins to play a greater role in skill improvement [11] at this stage.

Biological and social determinants of teaching and learning fundamental motor skills

Fundamental motor skills are best developed during childhood through a wide variety of exercises and movement experiences. They help to increase children's adaptive capabilities so that they are able to perform a wide range of motor tasks [12]. This process takes place alongside school education, during which three stages of motor development can be distinguished. Stage one (early middle childhood) begins at 7 and ends at 10 years of age. It is a period that is particularly beneficial in terms of teaching and learning fundamental motor skills. Due to the fact that children at this age learn new motor skills quickly and perform already acquired skills easily, this period is called 'the golden age' of development. It is characterized by high dynamics of motor development and proneness to movement acquisition. At this stage, children begin to manifest high levels of coordination between the limbs and

the trunk. At 8-10 years of age, the process of lateralization is finished. At this age, children become attracted to competition and their social as well as team-working skills are improved. At this stage, it is important to initiate situations that increase students' self-esteem to create opportunities for them to discover their capabilities and to promote social acceptance regarding different types of behaviour. When teaching motor skills, it is crucial to provide demonstration and limit external stimuli that can disturb the course of the performed activity. It is necessary to ensure that the activity in question is performed correctly [13, 14].

Stage two (late middle childhood) starts at 11 years of age and ends at the beginning of puberty. During this period, boys and girls demonstrate similar levels of motor abilities and skills. The acquisition of new and more complex skills is relatively fast. Therefore, tasks used for motor skill improvement can be more complex. At the age of 12, children may begin to lose interest in sport and physical activity [15]. Therefore, it is extremely important to regularly spark students' interest in those forms of physical activity which they find attractive. At this age, we should boost children's confidence and create opportunities for displaying responsible behavior.

Stage 3 occurs at puberty (adolescence). Puberty usually begins at the age of 12-13. It usually lasts for 3-4 years in girls and 5-6 years in boys. During this period, the so-called pubertal spurt takes place. It involves a sudden increase in body height and disproportionate growth of particular body parts. It may be accompanied by the lack of movement harmony; movements are clumsy, which leads to difficulties in performing some tasks. All these biomechanical changes may periodically affect the ability to learn new motor skills. Hormonal and emotional changes as well as mood swings and changes in interests and hobbies are also notable [14, 16]. Therefore, teachers should manifest great sensitivity and show understanding toward students at this age, because students at this stage of development want to be independent and strive to discover their own identity. When choosing forms of physical activity, teachers should often take into consideration individual preferences of students.

Teaching and learning fundamental motor skills

A common misconception is that learning fundamental motor skills occurs autonomously alongside progressing motor development. As a matter of fact, teaching any skill requires the consistent application of proper methods and exercises [17]. The effectiveness of teaching and learning fundamental motor skills during a PE class is affected by the time devoted to performing a given exercise, selection of methods and tools, delivery of suitable instructions and feedback, skillful manipulation of a student's focus of attention, giving a sense of agency and autonomy while performing exercises, ensuring proper organization of exercises, individualization of the learning process and adjustment of the level of difficulty to the level of a student's skills.

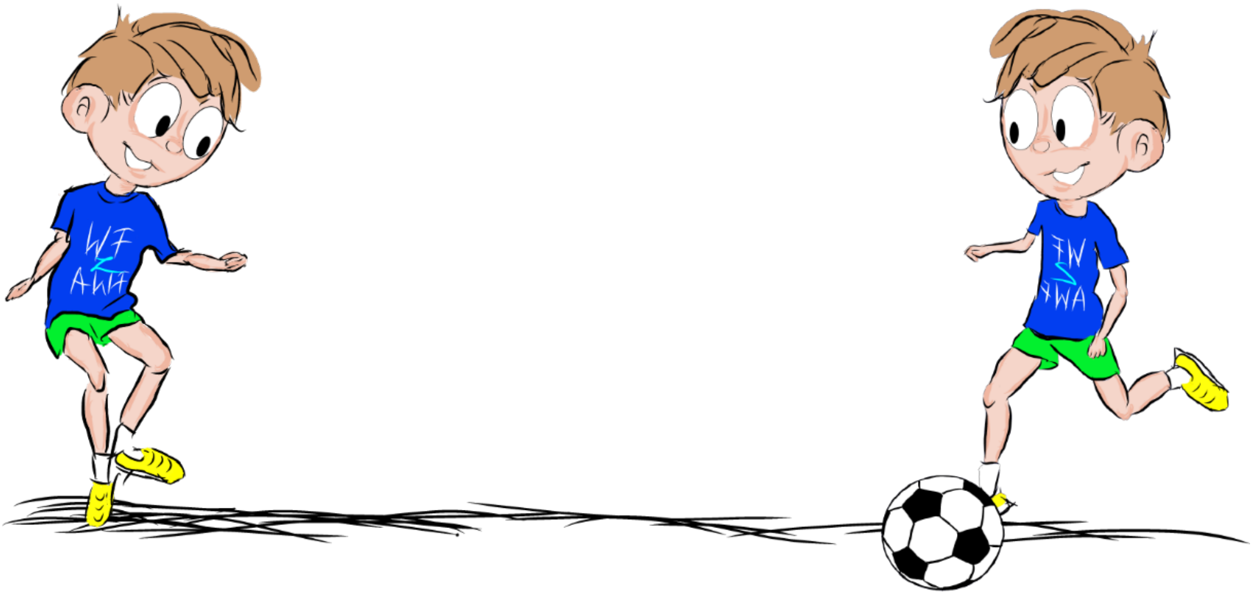

MAKE SURE THAT THE SKILL YOU TEACH CAN BE PERFORMED REPEATEDLY DURING A CLASS

It is important to avoid situations in which, due to the lack of equipment or too many people in a group, students wait too long for their turn to perform a task. Also, it is necessary to limit the use of full forms of sports in sport-specific conditions, since it prevents or seriously limits frequent repetitions of the skill which we want to improve. It is best to begin teaching a new skill in smaller groups, in pairs or even individually. Activity modifications should take into account a student's level of skill mastery. They can involve the use of various initial and final positions, the use of combinations of exercises performed symmetrically and asymmetrically, or to encourage students to explore a particular movement. Modifications can also be made by changing conditions in which exercises are performed by using non-typical accessories, apparatuses or surfaces as well as limiting visual control, time, physical space or including another student in the task [13]. Lastly, it must be emphasized that it is the teacher who drives the whole teaching process. Their attitude determines whether or not students participate in PE classes with pleasure and commitment. While teaching, it is worth praising students and making sure that after every class they have a sense of success. Teachers should evaluate the task performance and not students. It is important to provide students with feedback on errors in a competent way. Bearing in mind that students do not make errors on purpose, they should be told that errors are an indispensable part of any learning process. Information on errors should be given in a positive and vivid way so that students may know what can help them perform the task correctly.

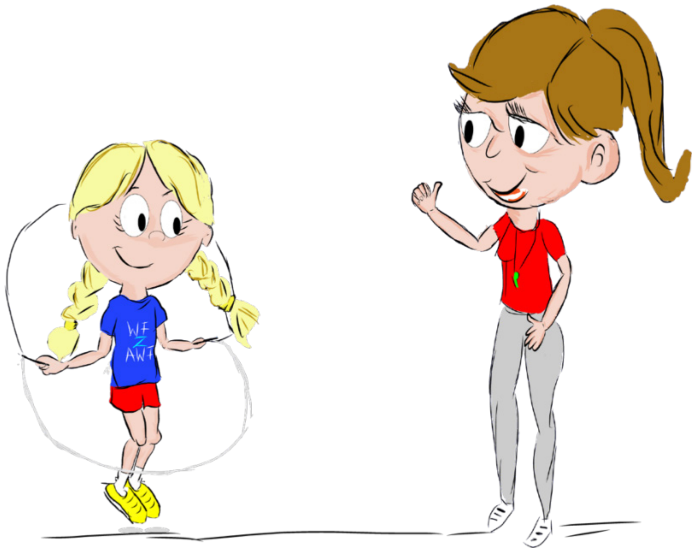

FEEDBACK ON TASK PERFORMANCE SHOULD BE POSITIVE AND SPECIFIC

Assessment of fundamental motor skills

A substantial role in the process of teaching and learning fundamental motor skills is played by tests and tools used for evaluating these skills. Early and accurate identification of deficits may reduce the risk of further delays in developing a particular task. Thus, the process of teaching and learning can be improved. When choosing a test, we should take into account how adequate it is to the needs defined in the PE program, what the target age group is, and what limitations stem from the lack of proper facilities and equipment. The most popular tests of sports skills include the Test of Gross Motor Development (TGMD-1, 2, 3), Motor Skill Checklist, Get Skilled Get Active, Basic Motor Competence (MOBAK), Canadian Agility and Movement Skill Assessment (CAMSA) and the Dragon Challenge (DC).

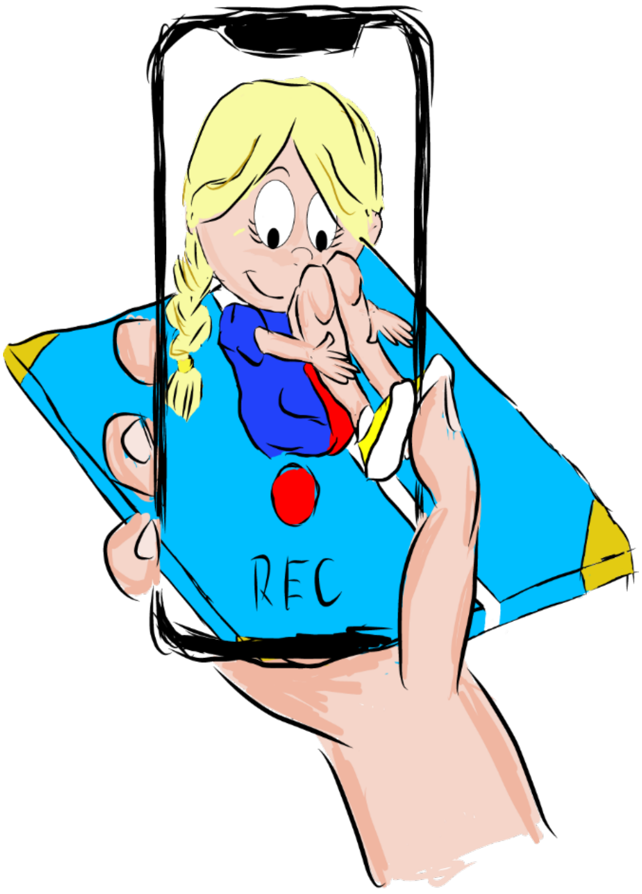

USE MODERN TECHNOLOGIES IN TEACHING AND LEARNING

Test of Fundamental Motor Skills in Sport (FUS)

A decreasing level of fundamental motor skills among students in many countries [18, 19] and infrequent monitoring of these skills point to the need for paying more attention to the process of teaching and learning these skills during PE classes. When it comes to applying tests of fundamental motor skills, a common barrier is the difficulty in carrying out an objective assessment based on an analysis of motor task performance. Another obstacle is the inappropriate adjustment of test tasks to movement-related needs of students, which stems from the lack of popularity of a given sport in a given country. A language barrier that makes it more difficult to conduct a test in another country may also constitute a limitation. Therefore, popularity of sports in a given country as well as universality and usefulness of particular motor skills in lifelong physical activity should constitute the basis for developing new fundamental motor skills. Moreover, a possibility of real, valid and reliable assessment of such skills should be taken into account. The above-mentioned guidelines were used as a guiding framework when developing assessments used in the Fundamental Motor Skills in Sport (FUS). The FUS test enables its users to assess the fundamental motor skills proficiency of students through the evaluation of six different motor (sports) tasks: hurdles, jumping rope, forward roll, ball bouncing, throwing and catching, kicking and stopping a ball.

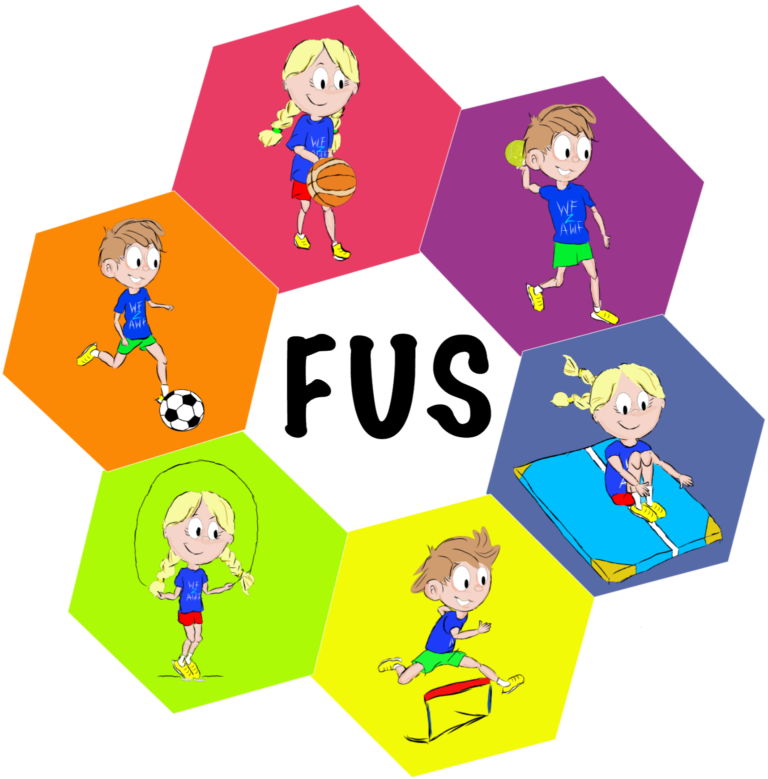

FUS test guidelines

Preparing the testing environment and equipment

The test venue and particular exercise stations should be prepared before the beginning of a PE lesson. The following equipment is needed to conduct the FUS test: hurdles or cardboard elements that imitate hurdles, jump ropes, a gym mat, basketballs, tennis balls, football (soccer) balls, cones, a measuring tape, a stopwatch, masking tape and a video recording device. Testing can be performed indoors but some tasks (or all of them) can be completed outdoors following detailed guidelines that can be found in the description of each task. The picture below is an example of how the exercise stations should be located in a sports gym. Students must wear shorts and T-shirts or any other well-fitting sports clothes that will make it possible to visually evaluate movements.

PREPARE THE FACILITY BEFORE STARTING THE TEST

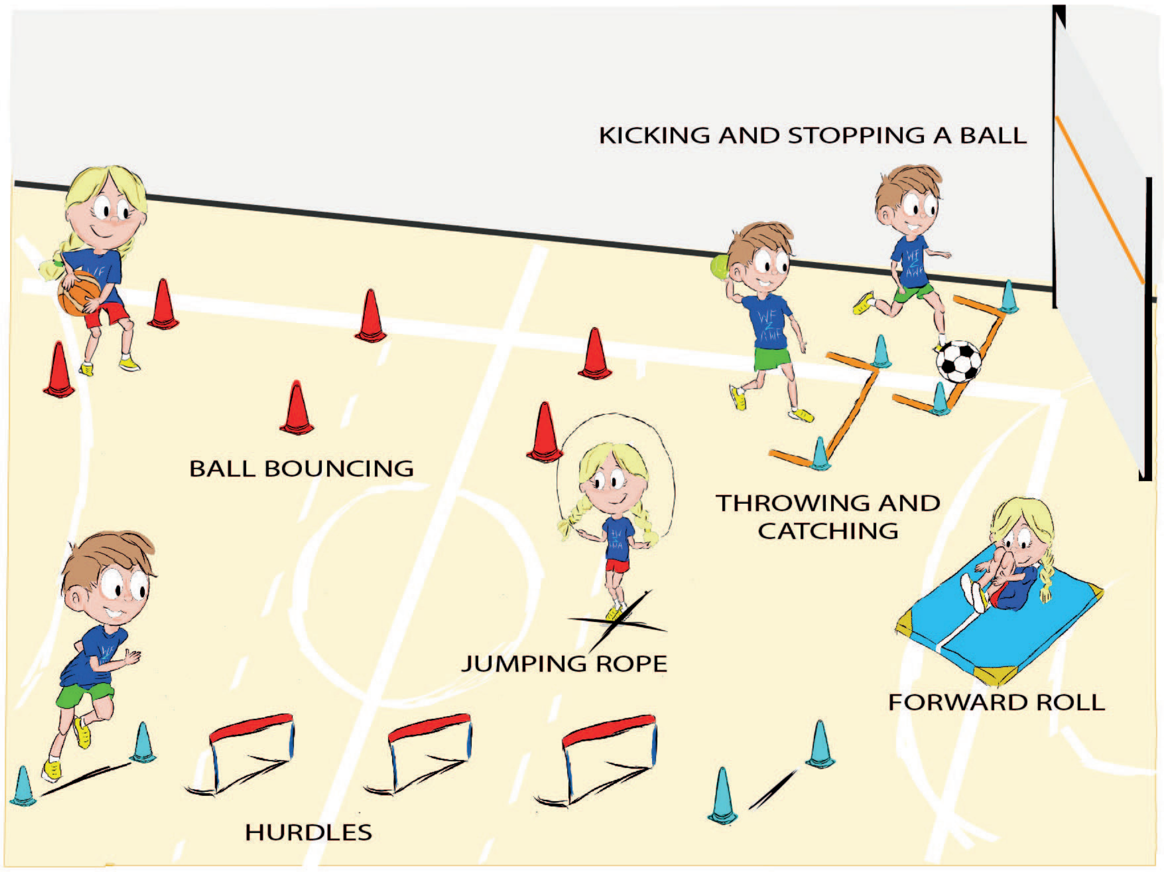

Test execution

The test should be preceded with a 5-6 minute general warm-up which includes running or movement games and dynamic stretching exercises. The warm-up is conducted by the teacher. Before each task, the teacher describes the activity and provides a demonstration how to perform the skill. On students' request, task instructions and performance can be repeated. The two test trials are preceded with a warm-up trial. Tasks such as hurdles, jumping rope, forward roll and ball bouncing are performed in two rounds. After all students complete the first round, they will all complete the second round of testing. In tasks like throwing and catching as well as kicking and stopping a ball, the second trial should be performed immediately following the first one.

Assessment guidelines

Assessment of a given skill is based on identifying the correctness of performing 5 components of this skill. Students get 1 point when a given component is performed according to the established criterion, while they get 0 points when the criterion is not met. Points are awarded only when the teacher is certain that a given criterion is met. The level of skill mastery is established based on the trial in which a student receives the highest number of points. If the score in both trials is the same, the score obtained in the first trial constitutes the final result. Task assessment (i.e., awarding points) is made after the testing procedure is completed. Assessment is carried out using software or a video player app. Video recordings should be replayed at a normal speed. If there are any doubts, slow motion analysis can be performed. In this case, it should be noted in the assessment sheet.

Criteria of assessing fundamental motor skills (FMS) proficiency

A fundamental motor skill in the FUS test is considered to be:

- ✓ **’full mastery’** when the student performed all skill components according to the established criteria (scored 5 points);
- ✓ **’near mastery’** when the student performed all but one component according to the established criteria (scored 4 points);
- ✓ **’some mastery’** when the student performed three skill components according to the established criteria (scored 3 points);
- ✓ **’poor’** when the student performed no more than two skill components correctly according to the established criteria (scored 2 points or fewer).

Overall FMS proficiency is assessed based on the level of mastery of particular motor skills. Four levels are distinguished:

- ✓ **’excellent FMS proficiency’** is obtained when the student has fully mastered all assessed skills (scored 5 points for each skill) or has mastered all but one skill at a ’near mastery’ level (received 4 points for this skill);
- ✓ **’good FMS proficiency’** is achieved when the student has reached at least the ’near mastery’ level for each assessed skill (scored at least 4 points for each skill) and has not met the requirements for ’excellent FMS proficiency’;
- ✓ **’elementary FMS proficiency’** is accomplished when the student has scored at the ’some mastery’ level for each assessed skill (scored at least 3 points for each skill) and has not met the requirements for ’excellent FMS proficiency’ or ’good FMS proficiency’.
- ✓ **’insufficient FMS proficiency’** is achieved when skill performance does not meet the requirements established for ’excellent FMS proficiency’, ’good FMS proficiency’, or ’elementary FMS proficiency’ levels.

References

1. Bardid F, Vannozzi G, Logan SW, Hardy LL, Barnett LM: A hitchhiker's guide to assessing young people's motor competence: Deciding what method to use. *Journal of Science and Medicine in Sport* 2019, 22(3): 311-318.
2. Lubans DR, Morgan PJ, Cliff DP, Barnett LM, Okely AD: Fundamental movement skills in children and adolescents. *Sports Medicine* 2010, 40(12): 1019-1035.
3. Stodden DF, Goodway JD, Langendorfer SJ, Roberton MA, Rudisill ME, Garcia C, Garcia LE: A developmental perspective on the role of motor skill competence in physical activity: An emergent relationship. *Quest* 2008, 60(2): 290-306.
4. Barnett LM, Van Beurden E, Morgan PJ, Brooks LO, Beard JR: Childhood motor skill proficiency as a predictor of adolescent physical activity. *Journal of Adolescent Health* 2009, 44(3): 252-259.
5. Cattuzzo MT, dos Santos Henrique R, Ré AHN, de Oliveira IS, Melo BM, de Sousa Moura M, de Araújo RC, Stodden D: Motor competence and health related physical fitness in youth: A systematic review. *Journal of Science and Medicine in Sport* 2016, 19(2): 123-129.
6. Burns RD, Bai Y, Byun W, Colotti TE, Pfledderer CD, Kwon S, Brusseau TA: Bidirectional relationships of physical activity and gross motor skills before and after summer break: Application of a cross-lagged panel model. *Journal of Sport and Health Science* 2020, 11(2): 244-251.
7. Macdonald K, Milne N, Orr R, Pope R: Relationships between motor proficiency and academic performance in mathematics and reading in school-aged children and adolescents: a systematic review. *International Journal of Environmental Research and Public Health* 2018, 15(8): 1603.
8. Rose E, Larkin D, Parker H, Hands B: Does motor competence affect self-perceptions differently for adolescent males and females? *Sage Open* 2015, 5(4): 2158244015615922.
9. Drummond M, Pill S: The role of physical education in promoting sport participation in school and beyond. *Youth sport in Australia: History and Culture* 2011: 165-178.

10. Barnett LM, Stodden D, Cohen KE, Smith JJ, Lubans DR, Lenoir M, Iivonen S, Miller AD, Laukkanen A, Dudley D: Fundamental movement skills: An important focus. *Journal of Teaching in Physical Education* 2016, 35(3): 219-225.
11. Fitts PM, Posner MI: *Human performance*. Belmont, CA: Brooks/Cole 1967, 5: 7-16.
12. Clark JE, Metcalfe JS: The mountain of motor development: A metaphor. *Motor Development: Research and Reviews* 2002, 2(163-190): 183-202.
13. Sozański H, Sadowski J, Czerwiński J: *Podstawy teorii i technologii treningu sportowego*. Tom II. Warszawa-Biała Podlaska: Akademia Wychowania Fizycznego Józefa Piłsudskiego w Warszawie, Filia w Białej Podlaskiej 2015.
14. Balyi I, Way R, Higgs C: *Long-term athlete development.* Human Kinetics 2013.
15. Robertson J: *Children, Aussie sports and organized junior sport: final report.* Canberra, Australia: Australian Sports Commission 1992.
16. Jurimae T, Jurimae J: *Growth, physical activity, and motor development in prepubertal children.* CRC Press 2001.
17. Magill R, Anderson D: *Motor learning and control.* New York: McGraw-Hill Publishing 2010.
18. O'Brien W, Belton S, Issartel J: Fundamental movement skill proficiency amongst adolescent youth. *Physical Education and Sport Pedagogy* 2016, 21(6): 557-571.
19. Farmer O, Belton S, O'Brien W: The relationship between actual fundamental motor skill proficiency, perceived motor skill confidence and competence, and physical activity in 8–12-year-old Irish female youth. *Sports* 2017, 5(4): 74.

Hurdles

Hurdles is the test that involves effective short-distance sprint running over hurdles. The skill of running over obstacles is directly used in a track and field event known as hurdles. The skills that are trained (rhythm of the run and controlling stride length) are crucial in effective performance of various types of running, track and field jumps, javelin throws and jumping in gymnastics. They also play an important role in set pieces in team sports (e.g., free kicks in football (soccer), kicks in rugby or American football, running shots in handball, and volleyball serves preceded with a run-up). Performing a run over hurdles may constitute the basis for assessing locomotor speed, whole-body movement coordination, dynamic balance, movement mobility as well as rhythm and movement coupling.

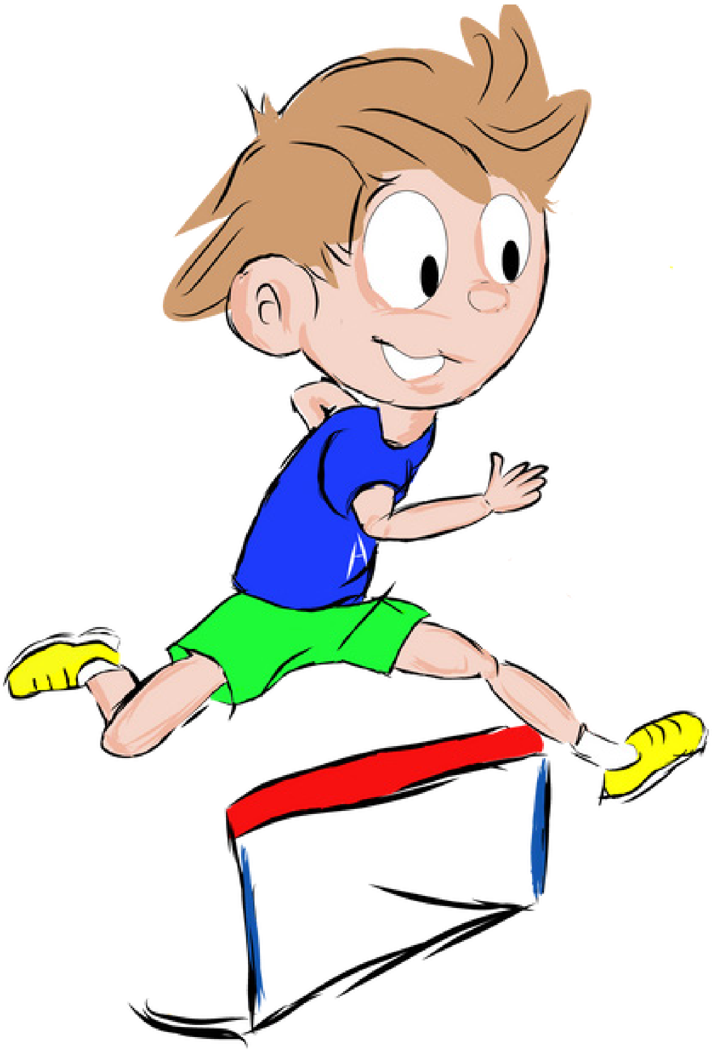

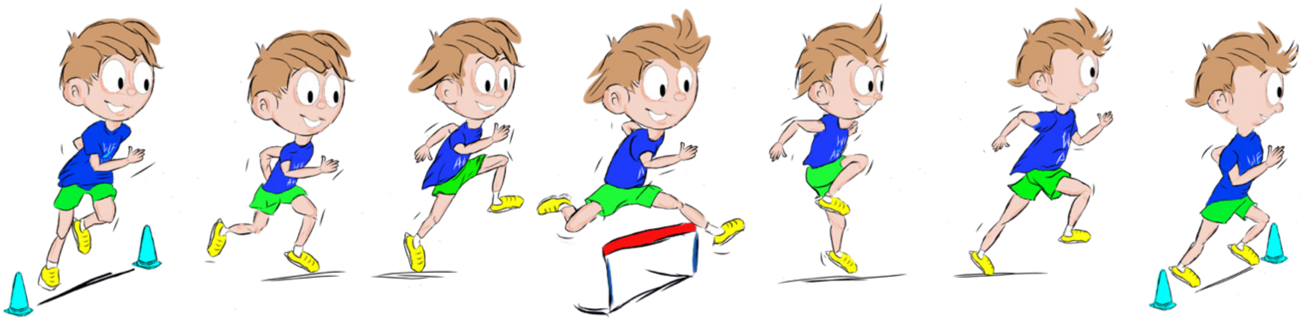

Student receives one point for each criterion met

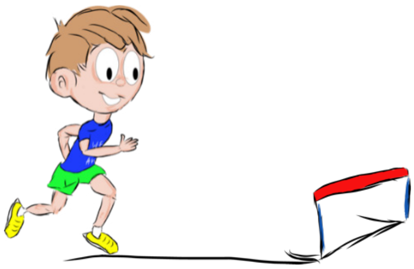

Criterion 1

The run-up (i.e., approach) to the first hurdle is fast, knees are lifted high and elbows are bent.

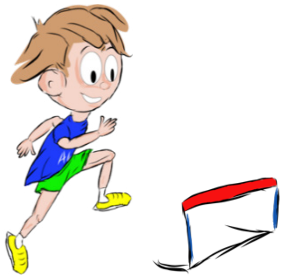

Criterion 2

There is no slow down prior to hurdle clearance, and there is clear forward movement during the take-off that precedes hurdle clearance.

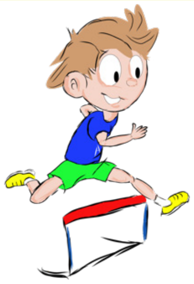

Criterion 3

Body moves flat over the hurdle, the trunk leans forward, the trail leg moves quickly forward (without stopping).

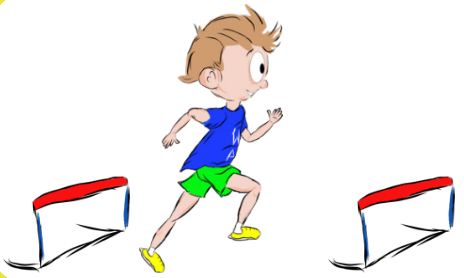

Criterion 4

Stride pattern between the hurdles is rhythmic, the number of strides between particular hurdles is the same.

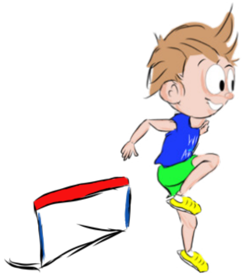

Criterion 5

There is no slow down after hurdle clearance, balance is maintained on landing and the run is continued in a straight line.

Test description and performance conditions

The task is to run over three hurdles (obstacles) in a 30 m run as fast as possible. The student is behind the start line in a standing start position, with one foot placed forward and the trunk slightly bent. The student begins their run at their convenience. The whole distance is covered running fast on the ball of the foot. All three hurdles are cleared using a simplified hurdling technique. Each hurdle is attacked with the lead leg slightly bent. On crossing over the hurdle, the student's lead leg snaps down quickly. The other leg (trail leg), which is bent at the knee, follows the lead leg quickly and smoothly. During the hurdle clearance, the student's trunk is bent slightly. The task is performed twice, with at least a 3-minute interval between the first and the second trial. The two trials are preceded with a warm-up trial.

The run is performed on a hard and flat surface either indoors or outdoors. The wind should be calm if the run is completed outdoors. The equipment used in the test includes hurdles or cardboard boxes that imitate hurdles. A minimum width of the hurdle is 0.8 m, while its height is adjusted to the students' age. Specifically, a height of 40 cm should be used for students aged 7-9, 50 cm should be used for students aged 10-12, and a height of 60 cm should be used for students aged 13-14. The distance from the start line to the first hurdle is 10 m, the distance between the hurdles is 7 m, and the distance between the last hurdle and the finish line is 6 m. A measuring tape should be used to ensure all heights and distances are accurate. The start line and the finish line are marked using cones.

Verbal instruction and demonstration

Instruction: 'Cover the distance as fast as possible, and go over each of the three hurdles as flat as you can'. Demonstration: The task is demonstrated by the test supervisor or the student who has already acquired this skill. During the demonstration, all students stand along the course with a clear view of the task.

Equipment

Three hurdles or cardboard boxes that imitate hurdles, 4 cones, a measuring tape.

Assessment

Assessment is made using a recorded film that is replayed at a normal speed. If there are any doubts about meeting the criteria, slow motion analysis can be performed. Alternatively, evaluation can be carried out immediately after performing the task using an assessment sheet.

Position of the video camera or the supervisor when recording the task

The camera, or supervisor, is positioned perpendicular to the line of the run, 6 m away from the second hurdle. The eye of the camera follows the running student.

Hurdles
(graphic representation)

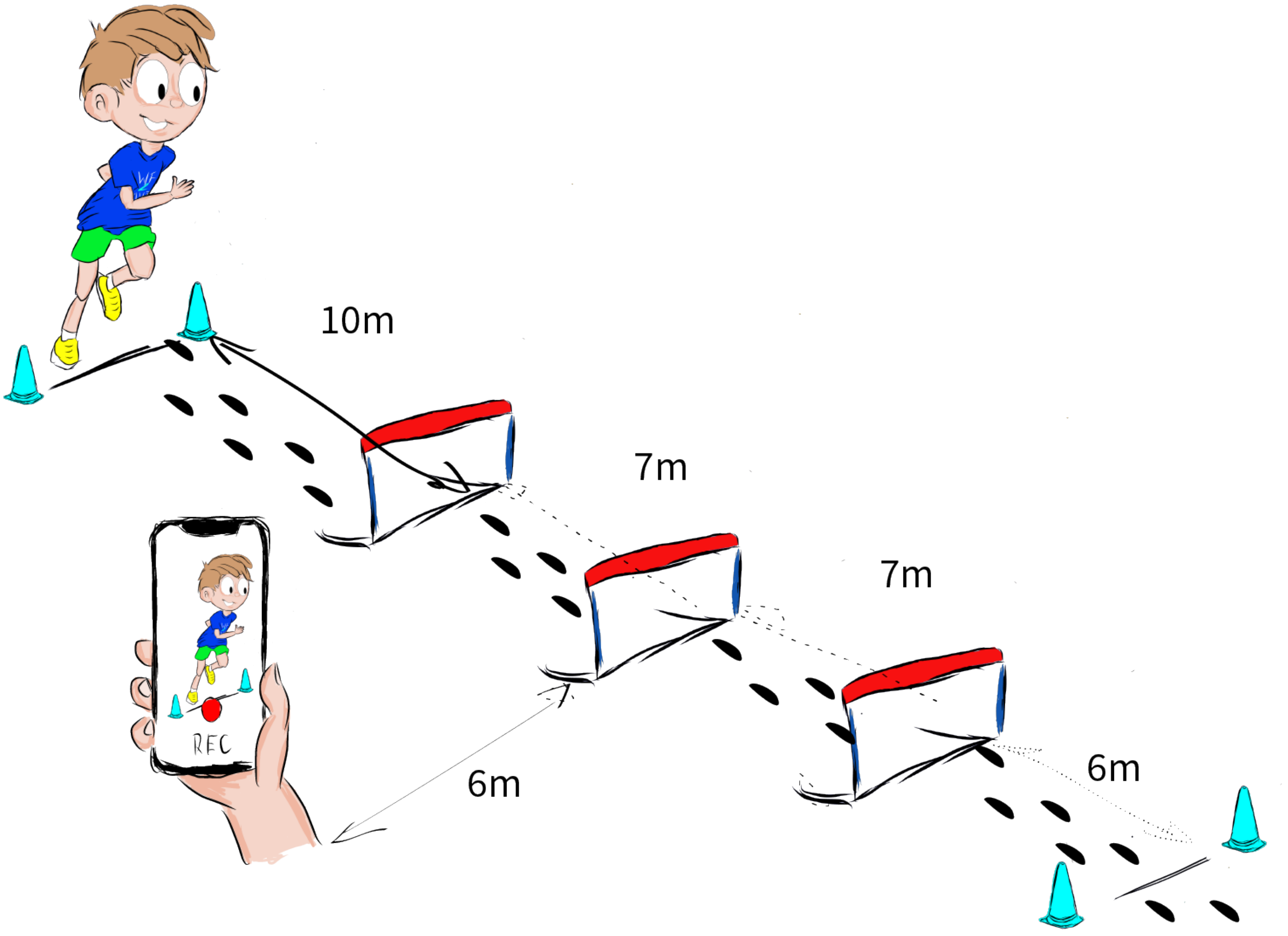

Jumping rope

Jumping rope is the test that involves performing rhythmic jumps over a rope that you pass over your head and under your feet. The skill of performing rhythmic jumps is used in many fun games and activities for children and adolescents, in various dance forms, fitness exercises and a wide range of whole-body exercises. This skill plays an important role in combat sports, gymnastics, team sports (e.g., volleyball or basketball) and other forms of physical activities in which movement coupling and rhythm are essential. Rope exercises are often used as a warm-up or health-oriented programs. Rope jump performance makes it possible to assess whole-body coordination, movement rhythm, jumping ability and dynamic balance.

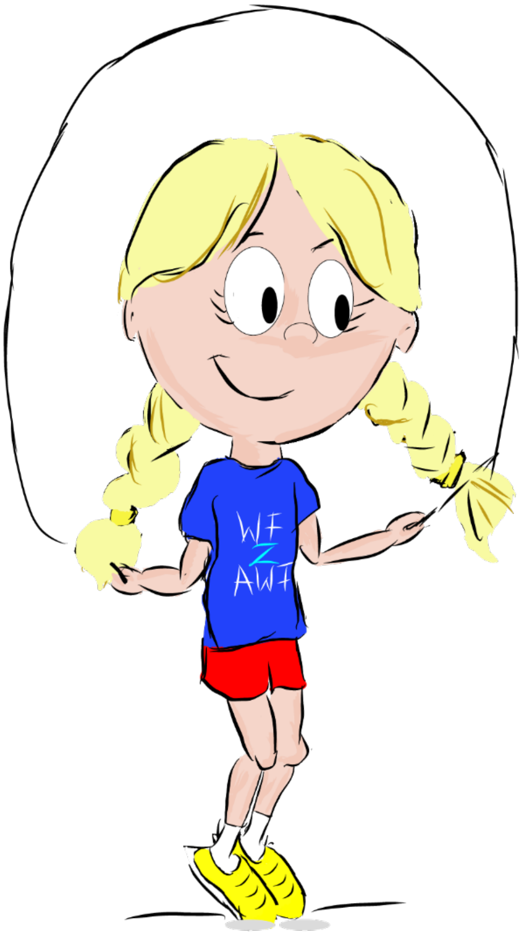

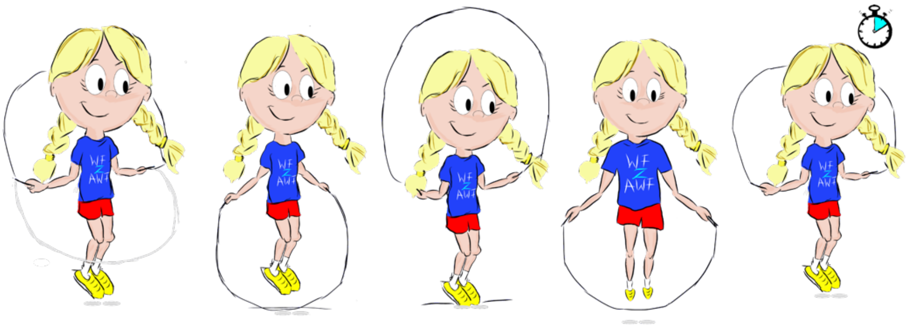

Student receives one point for each criterion met

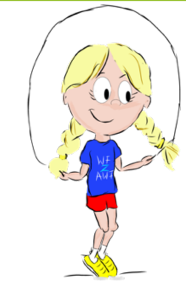

Criterion 1

Jumps are performed continuously (without stopping).

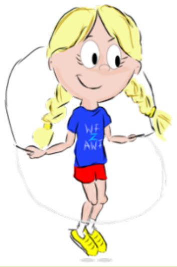

Criterion 2

Jumps are rhythmic and single, with short ground contact time and landing on the ball of the feet.

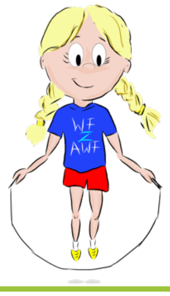

Criterion 3

Arms are bent and held close to the trunk, and the rope is moved using the rotation of forearms and wrists.

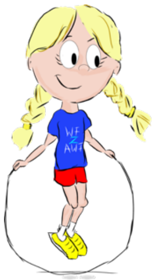

Criterion 4

Knees and hips are slightly bent during flight and landing.

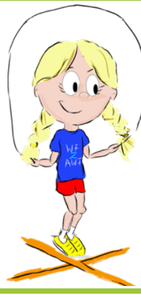

Criterion 5

Jumps are performed vertically with jumps initiating in the same designated area, with the trunk upright, feet parallel approximately hip width apart.

Test description and performance conditions

The task is to perform rhythmic and continuous jumps over the rope for 10 seconds. The student stands in the middle of the designated area formed by two 1-meter lines crossing each other at a right angle forming an X on the floor. Before commencing the task, the student stands in an upright position and holds the ends of the rope. The rope is behind the student. The arms are close to the trunk, elbows bent clearly and abducted externally. The student performs relatively low jumps, with knees and hips slightly bent during the flight and landing. The student takes off and lands on the ball of the foot. The trunk is held upright and feet are parallel (hip width apart). The task is performed twice, with at least a 3-minute interval between the first and the second trial. The main two trials are preceded with a warm-up trial.

Jumps are performed on a hard and flat surface. The length of the rope is adjusted to the student's body height. To verify the correct length, the rope is folded in half. With one end touching the ground beside the student, the other end should align with the student's shoulder.

Verbal instruction and demonstration

Instruction: 'Jump to the rhythm of the rope hitting the ground'. Demonstration: The task is demonstrated by the test supervisor or a student who has already acquired this skill. During the demonstration, all students stand directly in front of the task demonstrator.

Equipment

A jump rope, a stopwatch, a measuring tape and a masking tape.

Assessment

Assessment is made using a recorded film that is played at a normal speed. If any doubts about meeting the criteria arise, slow motion analysis can be performed. Alternatively, evaluation can be carried out immediately after performing the task using an assessment sheet.

Position of the video camera or the supervisor when recording the task

The camera, or supervisor, should be positioned facing the student performing the task, 4 m away from the center of the X on the floor.

Jumping rope
(graphic representation)

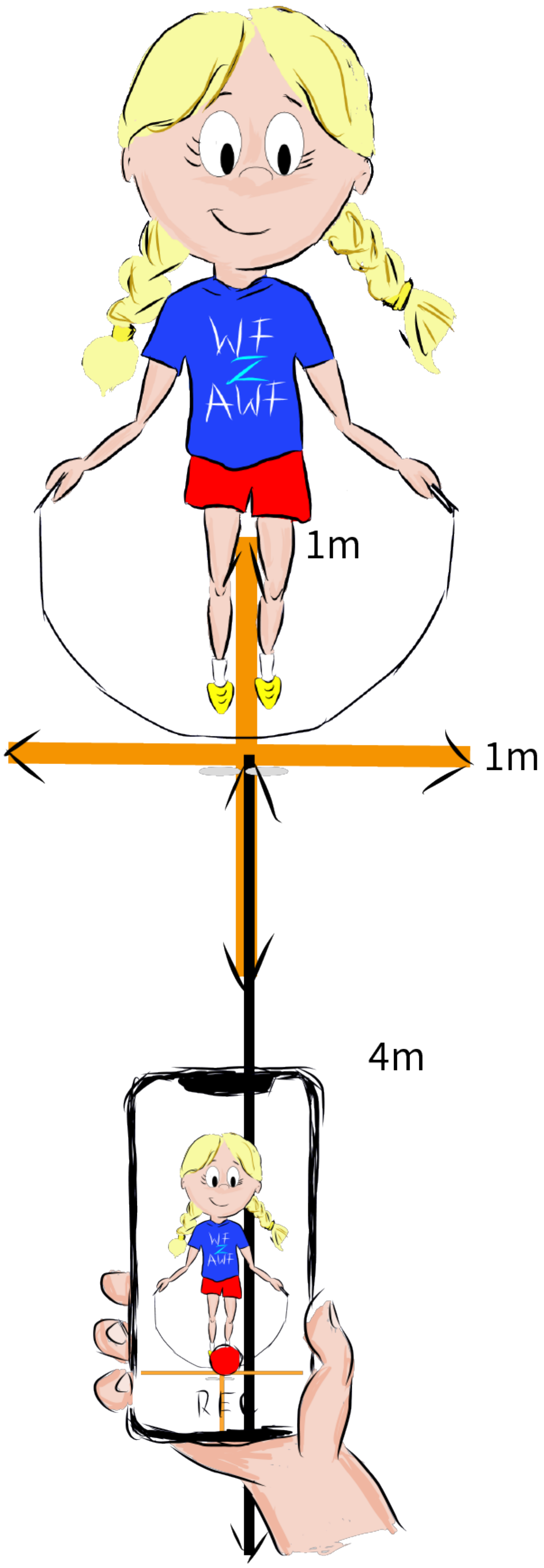

Forward roll

Performing a forward roll involves rolling over your back in a tucked position. The student begins and ends the forward roll in a squat position with hands placed on the mat or ground. This skill is used in gymnastics, combat sports (e.g., judo, wrestling) and some dance forms. The skill of performing the forward roll plays an important preventive role in all sports and physical activities in which there is a risk of falling or losing balance (e.g., cycling, rugby, handball, football (soccer), track and field). Moreover, the forward roll stimulates the vestibular system which is responsible for maintaining balance, receiving information related to gravitational force, maintaining proper muscle tone, maintaining constant visual field during head movements as well as movement planning. Performing the forward roll makes it possible to assess whole-body motor coordination, movement symmetry, spatio-temporal orientation, and dynamic balance.

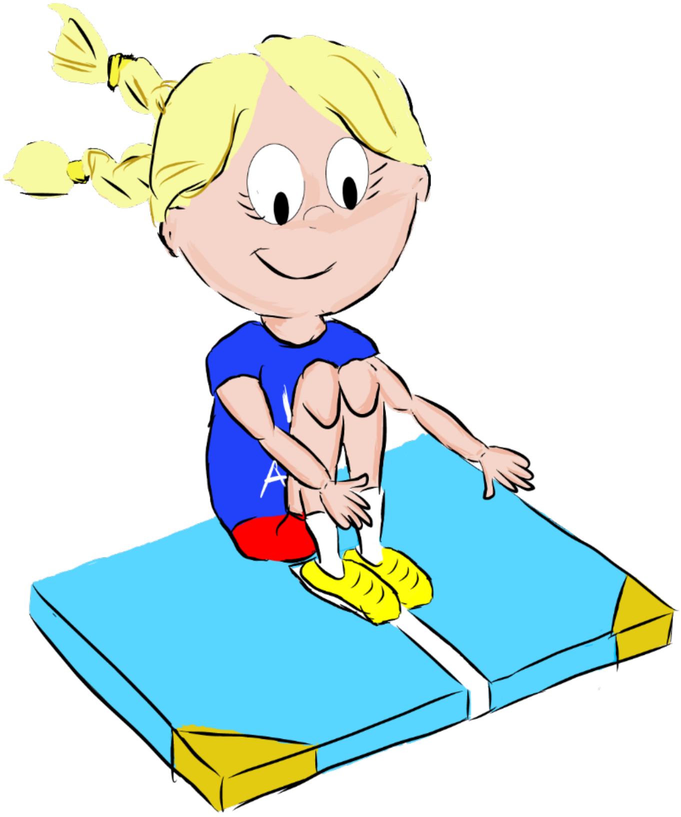

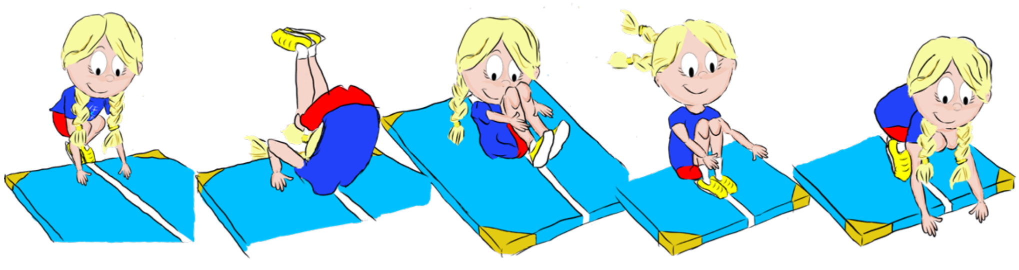

Student receives one point for each criterion met

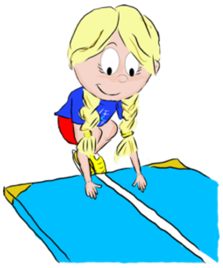

Criterion 1

The task is started in a squat position with both hands placed on the mat and the chin tucked into the chest; both legs are extended equally to push off the ground.

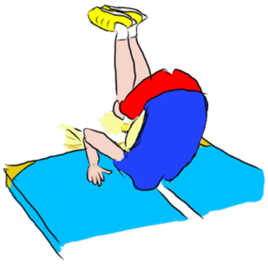

Criterion 2

Rolling over the back is performed without stopping and with the chin tucked.

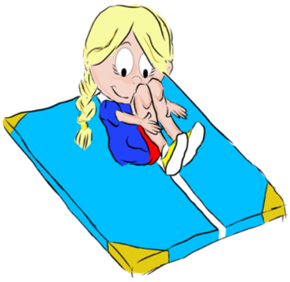

Criterion 3

Symmetry of movement is maintained while rolling, legs are bent and tucked to the chest.

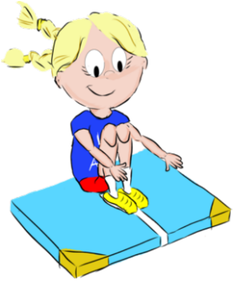

Criterion 4

Forward roll is performed in a straight line.

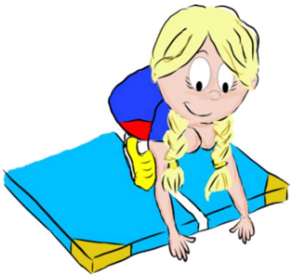

Criterion 5

The task is completed in a squat position with hands placed on the ground in front of the toes.

Test description and performance conditions

The task is to perform a forward roll starting and ending in a squat position with hands on the ground. First, the student performs the squat and places their hands on the mat. Then, the student raises the hips and extends both legs to push off the ground while tucking the chin. Next, body mass is transferred to the arms. With elbows bent, the student drops the head between the arms, places the neck on the mat and rolls forward with legs tucked to the chest. The task is completed in a squat position with hands placed on the ground in front of the toes. The task is performed twice, with at least 3-minutes between the first and the second trial. The two assessment trials are preceded with a warm-up trial.

The forward roll is performed on a gym mat of medium hardness. There is a line formed with a masking tape running through the center of the mat.

Verbal instruction and demonstration

Instruction: 'Perform a forward roll along the line'. Demonstration: The task is demonstrated by the test supervisor or a student who has already acquired this skill. During the demonstration, all students stand to the side facing the task demonstrator.

Equipment

A gym mat, a measuring tape, a masking tape that is a different color than the mat.

Assessment

Assessment is made using a recorded video that is played at a normal speed. If any doubts about meeting the movement criteria arise, slow motion analysis can be performed. Alternatively, evaluation can be carried out immediately after performing the task using an assessment sheet.

Position of the video camera or the supervisor when recording the task

The camera, or supervisor, is positioned at an angle of 45° on the left or right side of the student performing the task. The camera should be located at a distance of 3 m away from the nearest corner of the mat.

Forward roll
(graphic representation)

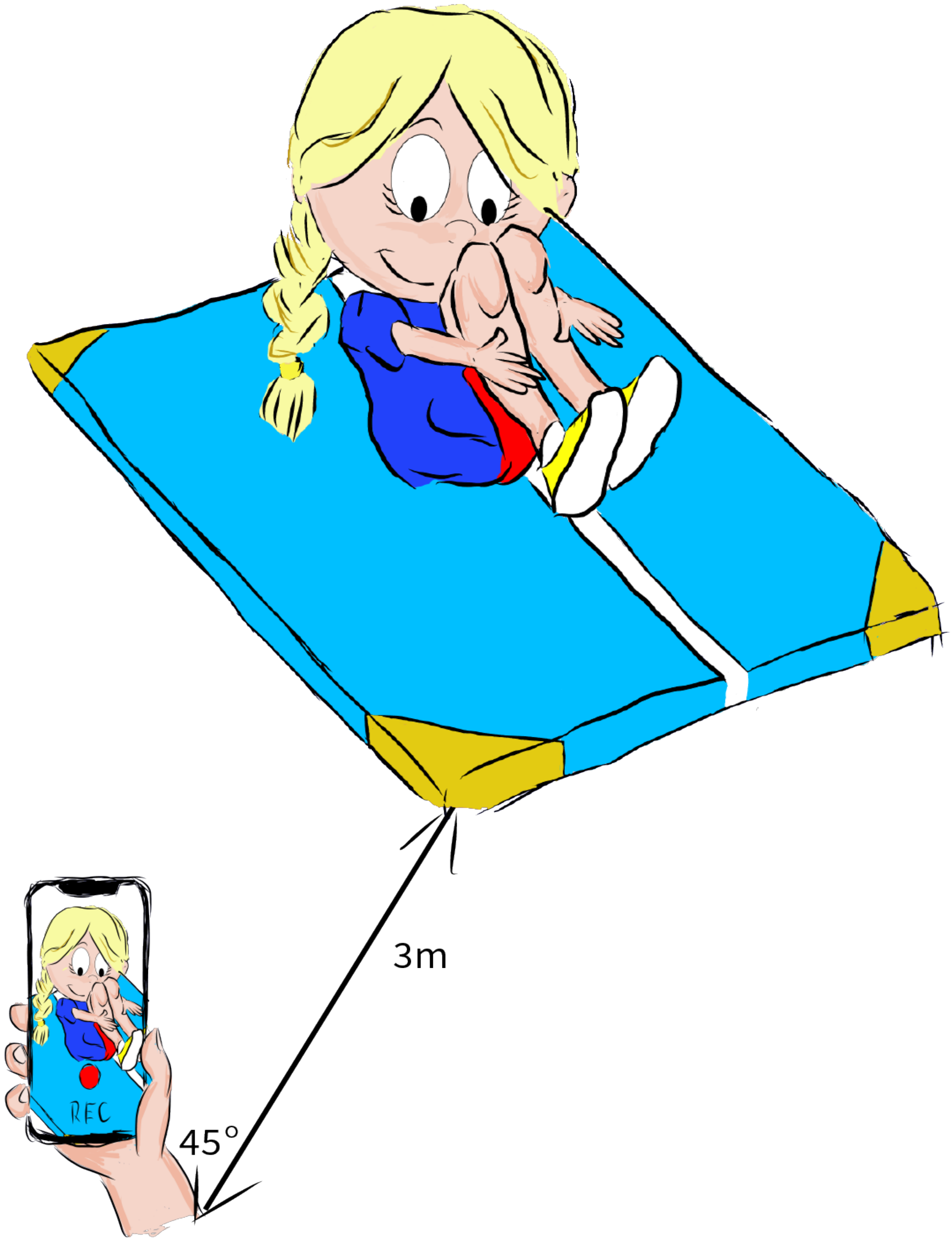

Ball bouncing

Ball bouncing (dribbling) is a test which involves bouncing a ball while walking and running. The skill of ball bouncing is necessary in basketball and handball. It is also used in all ball sports in which it is important to be able to control a ball (e.g., as an activity that precedes a tennis or volleyball serve). Bouncing a ball while walking and running helps to assess agility, whole-body coordination with a special focus on eye-hand coordination, timing, movement coupling, kinesthetic ability and peripheral vision.

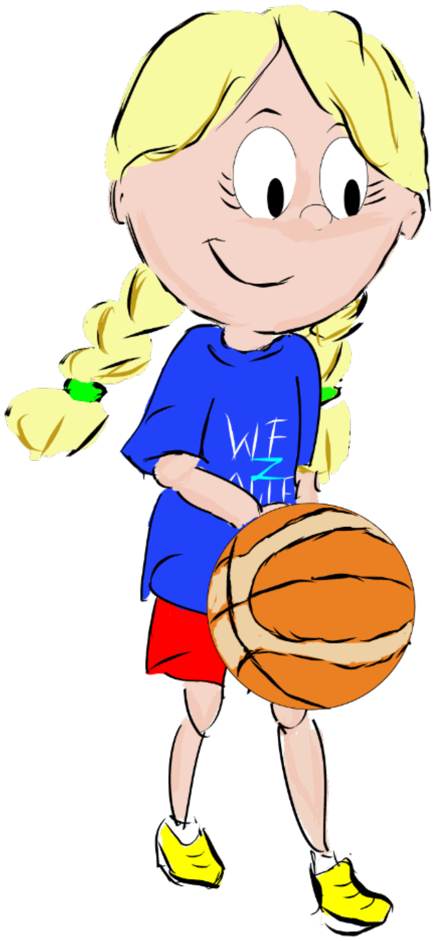

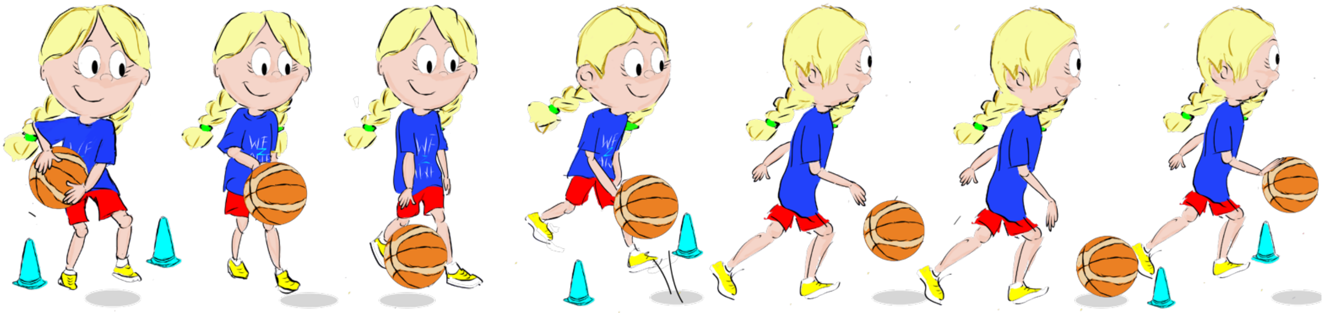

Student receives one point for each criterion met

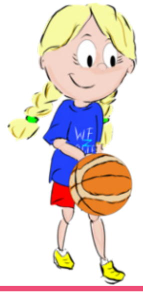

Criterion 1

In the first 10 m of the test the ball is rhythmically bounced at hip height with the top of the ball remaining below the chest while walking in a straight line.

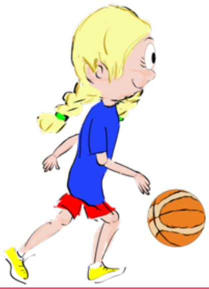

Criterion 2

The second 10 m of the test is running and bouncing the ball with the ball remaining relatively close to the body.

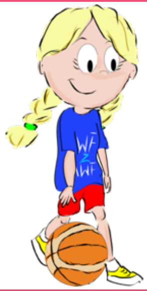

Criterion 3

The whole distance (20 m) is covered bouncing the ball in front of and slightly to the side of the body. The ball is not carried during any time throughout the test.

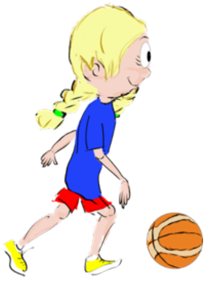

Criterion 4

The elbow and wrist are extended when the ball is pushed toward the ground. The ball is controlled with the tips of the fingers.

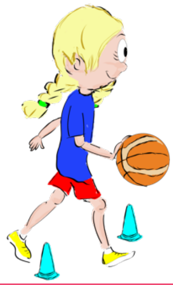

Criterion 5

The trunk is upright while the ball is bounced (students aged 7-9) and for students aged 10-14, eyes are focused forward while the ball is bounced.

Test description and performance conditions

The task is to bounce the ball while walking for 10 m and running an additional 10 m, for a total distance of 20 m. The student begins performing the task in an upright stance with knees slightly bent, holding the ball with two hands and facing the direction of movement. The first part of the distance is covered by walking and bouncing the ball, while the second part is covered while running and bouncing the ball. Throughout the task, the ball is bounced at hip height, in front of and slightly to the side of the body. Bouncing is performed with the dominant hand. While bouncing, the ball is clearly pushed toward the ground by extending the elbow and the wrist. The head is held vertically with the eyes looking forward. The task is performed twice, with at least a 3-minute interval between the first and the second trial. The main two assessment trials are preceded by a warm-up trial.

The task is performed on a hard and flat surface. The whole distance of 20 m is divided into two equal parts that are marked with cones at 0, 10 and 20 m. The width of the lane is 1.5 m.

Verbal instruction and demonstration

Instructions for students aged 7-9: 'Bounce the ball at hip height, while walking and then running. Keep your body upright'. Instructions for students aged 10-14: 'Bounce the ball at hip height, while walking and then running. Keep your eyes focused forward'. Demonstration: The task is demonstrated by the test supervisor or a student who has already acquired the skill. During the demonstration, all students stand along the lane facing the task demonstrator.

Equipment

Number 5 sized basketballs should be used for boys and girls aged 7-9 and girls aged 10-12. Number 6 sized basketballs should be used for boys aged 10-14, and girls aged 13-14. Additionally, the test requires 6 cones, masking tape, and a measuring tape.

Assessment

Assessment is made using a recorded film that is replayed at normal speed. If any doubts about meeting the criteria arise, slow motion analysis can be performed. Alternatively, the evaluation can be carried out immediately after performing the task using an assessment sheet.

Position of the video camera or the supervisor when recording the task

The camera, or the supervisor, should be positioned perpendicular to the line of the run, 15 m away from the start line and 5 m away from the side of the lane determined by cones which are situated closer to the camera. The camera should follow the student as they travel the 20 m of the test.

Ball bouncing
(graphic representation)

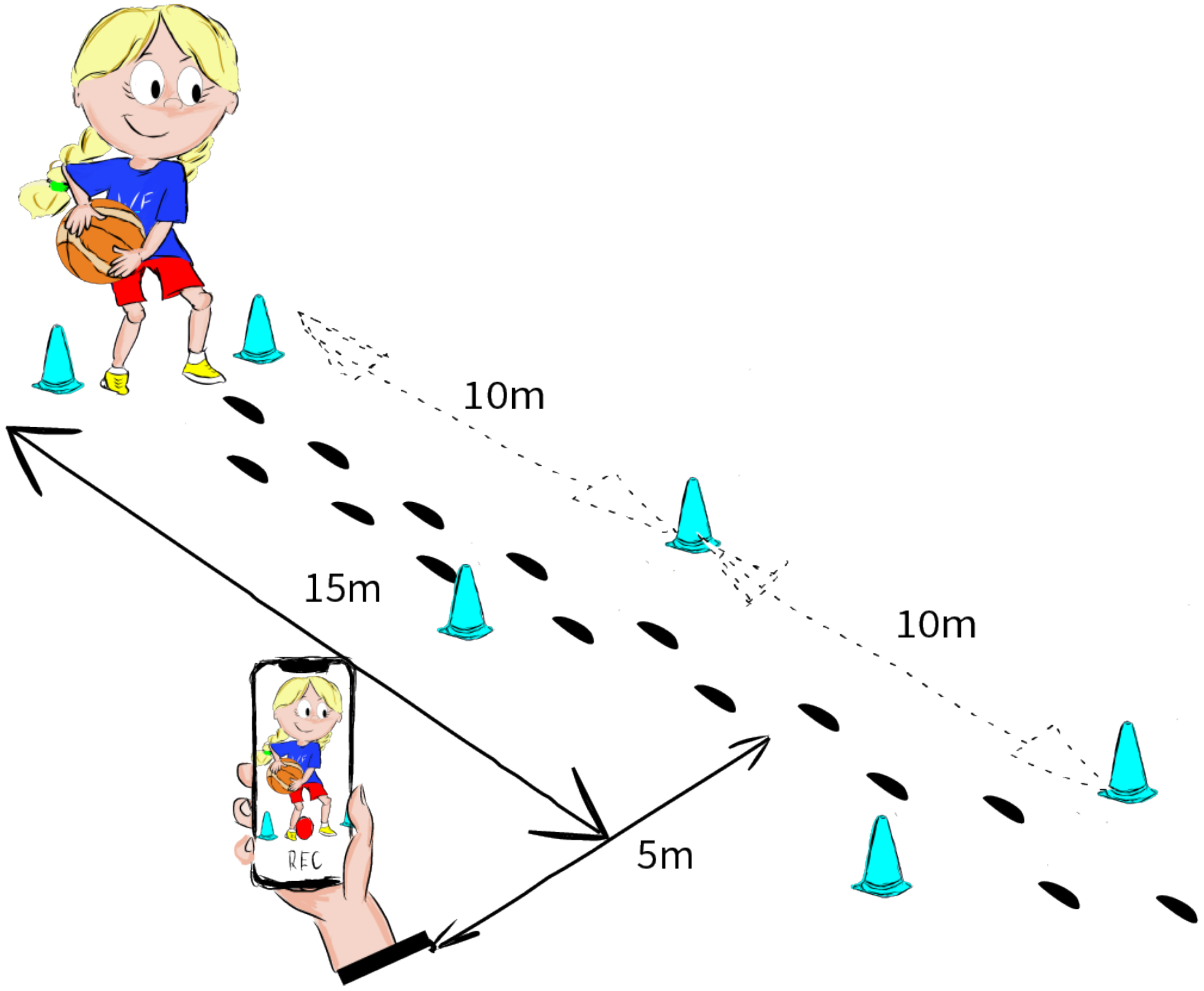

Throwing and catching

Throwing and catching a ball involves letting go of the ball in an intended direction following the motion of the hand and arm using a one-handed overhead throwing motion and catching it with one or both hands after it bounces against the wall. The throw is preceded with a run-up. The skill of throwing and catching the ball is a fundamental skill used in many sports such as baseball, basketball, handball, football (soccer), korfball, rugby, American football and softball as well as in a number of fun games and activities played by children and adolescents. The test helps to evaluate whole-body coordination, eye-hand coordination, movement coupling, spatio-temporal orientation, kinesthetic ability and throwing accuracy.

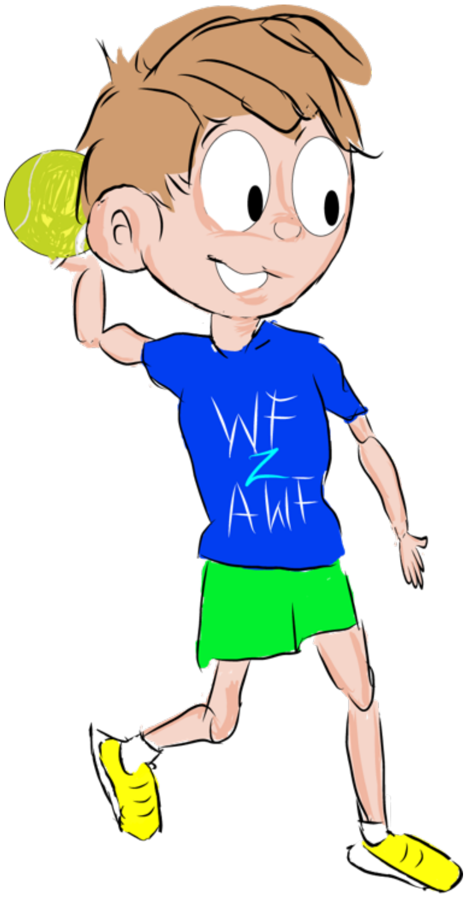

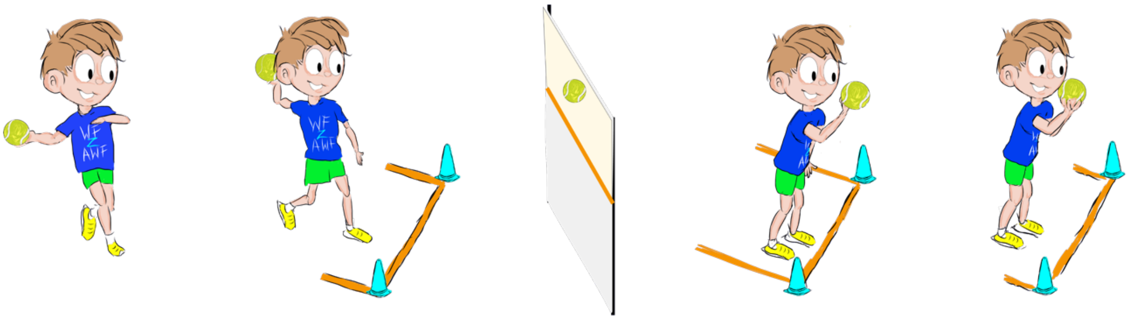

Student receives one point for each criterion met

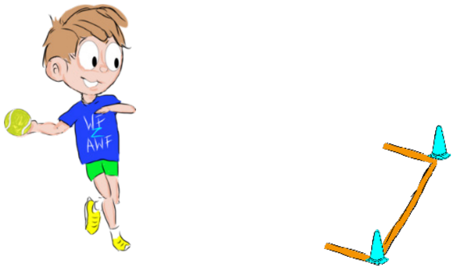

Criterion 1

The run-up is performed continuously without crossing the line marked on the floor.

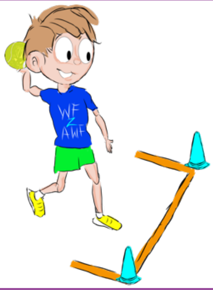

Criterion 2

The throw is initiated with the throwing arm is brought back and the foot of the opposite leg is clearly in front of the body; afterward, the overhead throw is performed.

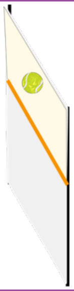

Criterion 3

The ball hits the wall above the line (in the target area).

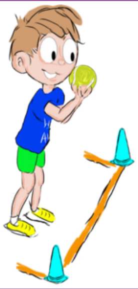

Criterion 4

The ball is caught, and hands do not touch the chest.

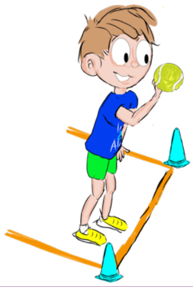

Criterion 5

The student remains behind the designated line when catching the ball.

Test description and performance conditions

The task is to perform a one-handed overhead throw with a run-up, hit the targeted area of the wall with the ball, and then catch the ball with one or both hands after it bounces against the wall. The student begins performing the task in an upright stance. After a 3-4-stride run-up, the student adopts the throwing position with the hand brought back, the trunk and hips are angled towards the target area, the foot of the opposite leg to is in front of the body. Then, the student rotates the hips in the direction of the throw followed by the rotation of the shoulders. The ball then moves close to the head, with the ball then being thrown toward the wall. After the throw, the trail leg is moved to the front without crossing the line marked on the floor. The throw is performed using the dominant arm. After the ball bounces against the wall, the student catches it in the designated area in an upright stance facing the wall. Students aged 7-9 should catch the ball using both hands immediately after it bounces against the wall or after it bounces against the wall and against the floor. Those aged 10-12 should catch the ball with both hands after it bounces against the wall, while those aged 13-14 should catch the ball with one hand after it bounces against the wall. The task is performed twice, with one trial directly following the another. The two assessment trials are preceded with a familiarization trial.

The throw is performed without crossing the line marked on the floor. The distance between the line and the wall is 5 m for students aged 7-12 and 6 m for those aged 13-14. The target area on the wall is above a line 3 m in length that is situated at the height of 3 m above the floor. At both ends of the 3 m line there are 1-metre lines that are perpendicular to the main horizontal line. The designated area for catching the ball is a 3 x 3 m square marked on the floor. Taped lines and cones are used to mark the parameters of the square.

Verbal instruction and demonstration

Instruction: ‘Throw the ball at the wall above the line so you can catch it’. Demonstration: The task is demonstrated by the test supervisor or a student who has already acquired the skill. During the demonstration, all students stand to the side facing the task demonstrator.

Equipment

Tennis balls, 4 cones, measuring tape, masking tape, a gymnastics box (ladder).

Assessment

Assessment is made using a recorded film that is replayed at a normal speed. If any doubts about meeting the criteria arise, slow motion analysis can be performed. Alternatively, evaluation can be carried out immediately after performing the task using an assessment sheet.

Position of the video camera or the supervisor when recording the task

The camera, or the supervisor, is positioned behind the student at an angle of 45° on the left or right side of the student performing the task. The throw should be recorded or observed at a distance of 5 m away from the middle of the main line marked on the floor.

Throwing and catching
(graphic representation)

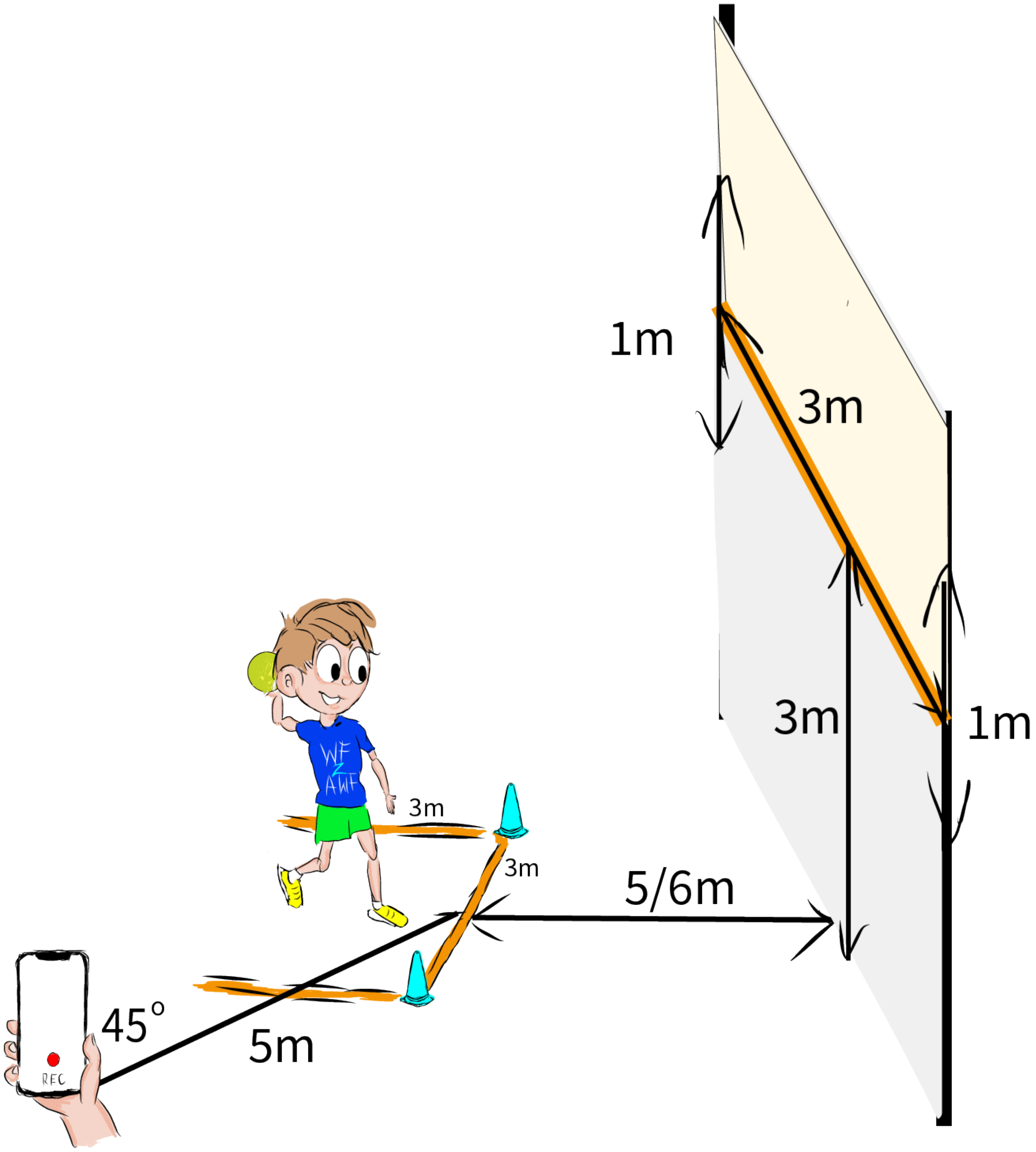

Kicking and stopping a ball

Kicking and stopping a ball involves kicking the ball with the foot, hitting the target area marked on the wall, and stopping the returning ball with the foot. Kicking the ball is preceded with a run-up. The skill of kicking and stopping a ball is necessary in football (soccer), kicking in rugby, American football, futnet, as well as in many fun games and activities for children and adolescents. The test assesses whole-body coordination, movement coupling, spatio-temporal orientation, kinesthetic ability, and kicking accuracy.

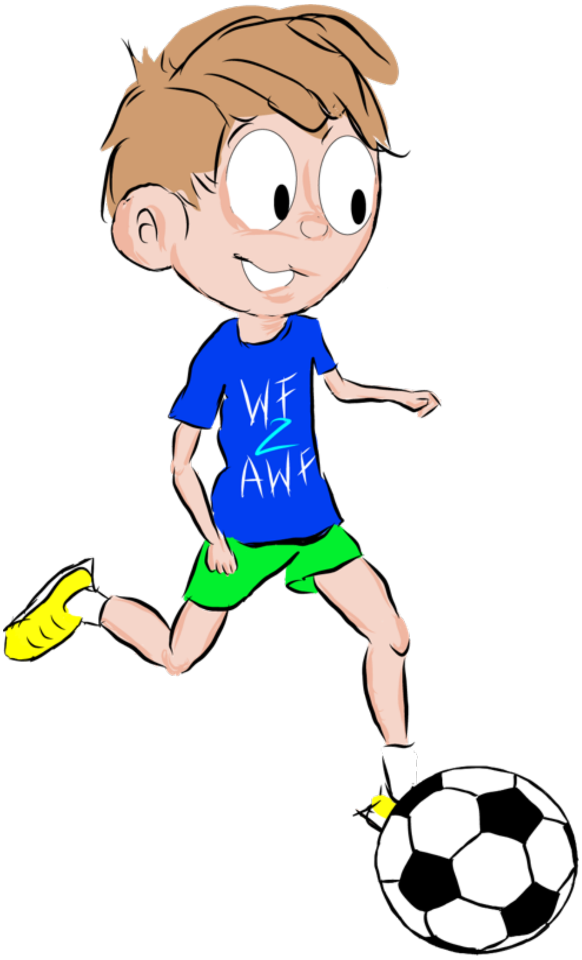

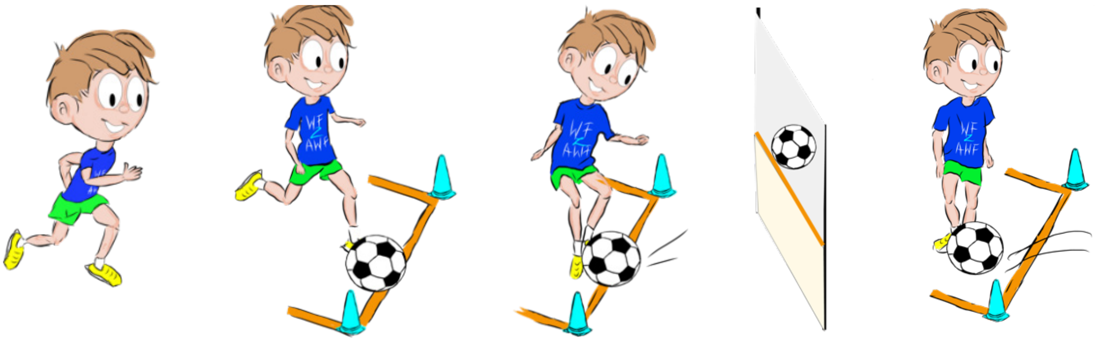

Student receives one point for each criterion met

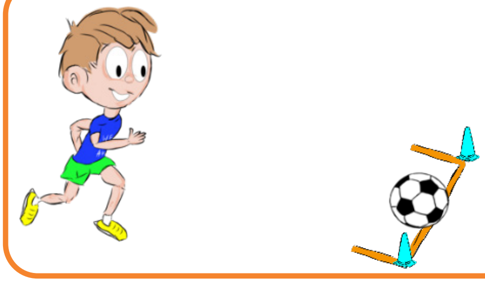

Criterion 1

The run-up is performed continuously, and the line marked on the floor is not crossed following the kick.

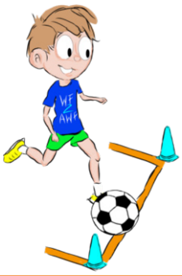

Criterion 2

The kicking leg is bent at the knee during the backswing for the kick, the non-kicking foot is placed beside the ball.

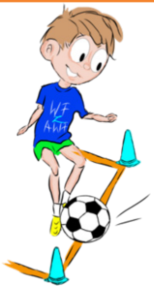

Criterion 3

The ball is kicked with the instep, top, or the side of the foot.

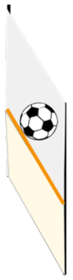

Criterion 4

The ball hits the target area marked on the wall, returns immediately to the student, and crosses the line of the designated area marked on the floor.

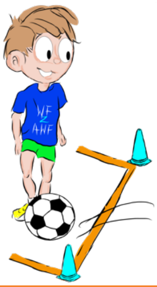

Criterion 5

After hitting the target area, the ball is stopped with one foot in the designated area.

Test description and performance conditions

The task is to run-up and kick (i.e., direct) the ball with the foot toward the target area and hit the target area marked on the wall, and to stop the returning ball with the foot. The student begins the task in an upright stance. After a 3-4-stride run-up, the student places the non-kicking foot beside the ball and kicks it with the instep, top, or side of the foot. The ball is directed to the target area on the wall. The kick is performed with the dominant leg. After the ball bounces against the wall, the student stops it with the foot in the designated area. The task is performed twice with one trial being performed immediately after the other. The two assessment trials are preceded with a warm-up trial.

The kick is performed without crossing the line marked on the floor. The line is situated 5 m away from the wall for students aged 7-12 and 6 m for students aged 13-14. The ball is situated in front of and in the middle of the line marked on the floor, in a 5 x 5 cm square formed with masking tape. Students aged 7-9 kick the ball toward a 3 x 3 m square marked on the wall. The floor constitutes a lower side of the square. Students aged 10-14 kick the ball toward a 3 x 2.5 m square (3 m high and 2.5 m wide) on the wall. The lower side of the square is marked with the line situated 0.5 m above the floor.

Verbal instruction and demonstration

Instruction: 'Kick the ball so that it hits the target area on the wall. Once the ball returns to you, stop it with your foot.' Demonstration: The task is demonstrated by the test supervisor or a student who has already acquired the skill. During the demonstration, all students should stand at the side facing the task demonstrator.

Equipment

Number 4 sized football (soccer) balls for students aged 7-9 and number 5 sized football (soccer) balls for students aged 10-14, 4 cones, masking tape, and a measuring tape.

Assessment

Assessment is made using a recorded video that is replayed at normal speed. If any doubts about meeting the criteria arise, slow motion analysis can be performed. Alternatively, evaluation can be carried out immediately after performing the task using an assessment sheet.

Position of the video camera or the supervisor when recording the tas

The camera, or the supervisor, is positioned behind the student performing the task at an angle of 45° to the left or right side, 5 m away from the middle of the line marked on the floor.

Kicking and stopping a ball
(graphic representation)

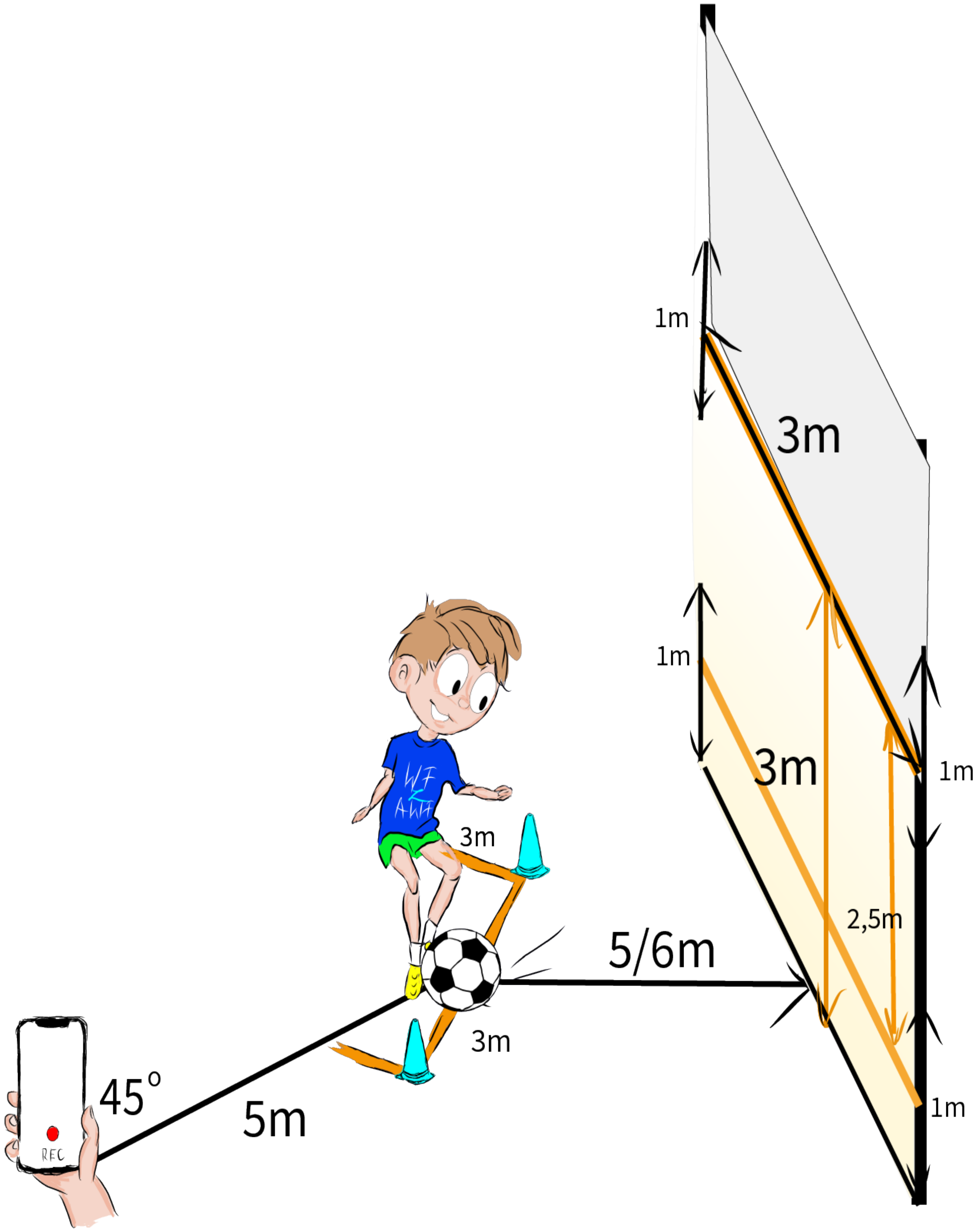

Hurdles (shortened criteria sheet)

Student's full name	**CRITERION 1** the run-up to the first hurdle is fast	**CRITERION 2** no slow down prior to hurdle clearance, clear forward movement during take-off	**CRITERION 3** body moves flat over the hurdle	**CRITERION 4** rhythmic stride pattern between hurdles	**CRITERION 5** no slow down after hurdle clearance

Notes:

Jumping rope (shortened criteria sheet)

Student's full name	**CRITERION 1** jumps are performed continuously	**CRITERION 2** jumps are rhythmic and single, they are performed on the balls of the feet	**CRITERION 3** arms are held close to the trunk, clear rotation of forearms	**CRITERION 4** knees and hips are slightly bent during flight/landing	**CRITERION 5** jumps are performed vertically from the same start location

Notes:

Forward roll (shortened criteria sheet)

Student’s full name	**CRITERION 1** the task is started in a squat position and with two-legged take-off	**CRITERION 2** rolling over the back without stopping	**CRITERION 3** symmetry of movement while rolling, legs are tucked to the chest	**CRITERION 4** forward roll is performed in a straight line	**CRITERION 5** the task is completed in a squat position with hands placed on the ground

Notes:

Ball bouncing (shortened criteria sheet)

Student's full name	**CRITERION 1** the ball is bounced at hip height while walking	**CRITERION 2** the ball is bounced close to the body while running	**CRITERION 3** the ball is bounced in front and to the side of the body	**CRITERION 4** the arm is extended when the ball is bounced	**CRITERION 5** eyes are focused forward, the trunk is upright

Notes:

Throwing and catching (shortened criteria sheet)

Student's full name	**CRITERION 1** continuous run-up without crossing the line	**CRITERION 2** throwing arm and opposite leg are contralateral before the throw	**CRITERION 3** the ball hits the wall above the line, in the target area	**CRITERION 4** the ball is caught and hands do not touch the chest	**CRITERION 5** the designated area is not left when catching the ball

Notes:

Kicking and stopping a ball (shortened criteria sheet)

Student’s full name	**CRITERION 1** continuous run-up without crossing the line	**CRITERION 2** the foot is placed beside the ball before kicking	**CRITERION 3** the ball is kicked with the instep of the foot	**CRITERION 4** the ball hits the target area and returns immediately	**CRITERION 5** the ball is stopped with the foot

Notes:

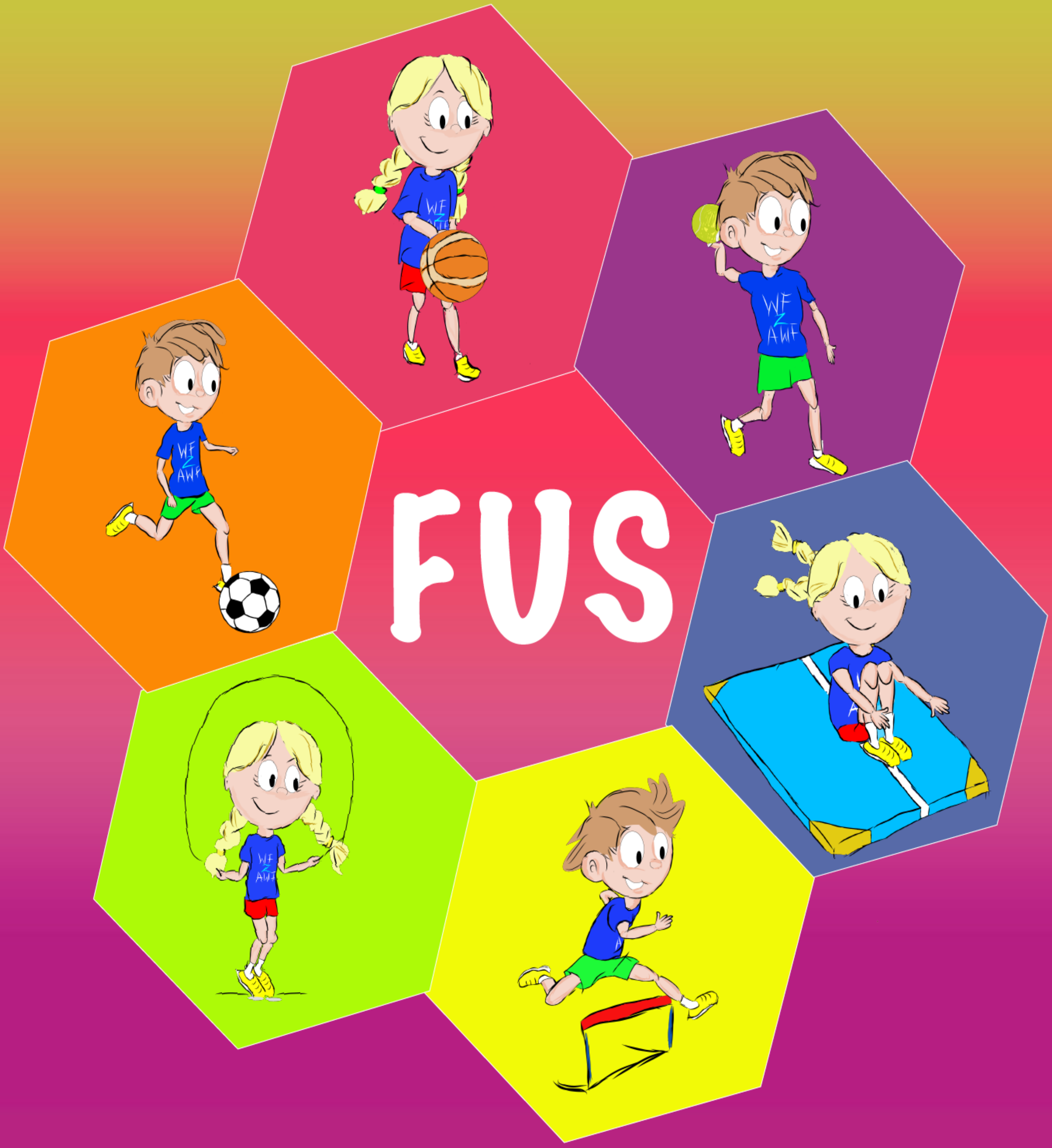
FUS
